# Comprehensive Multiomic Analysis Identified TUBA1C as a Potential Prognostic Biological Marker of Immune-Related Therapy in Pan-Cancer

**DOI:** 10.1155/2022/9493115

**Published:** 2022-10-30

**Authors:** Yiming Zou, Guoqing Wang, Min Fan

**Affiliations:** ^1^Department of Urology Surgery, The Third Affiliated Hospital of Soochow University and The First People's Hospital of Changzhou, Changzhou, Jiangsu 213000, China; ^2^Department of General Surgery, The Third Affiliated Hospital of Soochow University and The First People's Hospital of Changzhou, Changzhou, Jiangsu 213000, China

## Abstract

TUBA1C is correlated with an unfavourable prognosis and the infiltration of immune cells in several cancers. However, its function as a significant biomarker for the prognosis of immunotherapy in pan-cancer remains unclear. This study aims at assessing the role of TUBA1C in pan-cancer at multiple levels, including mutations, gene expression, methylation, m^6^A methylation, and immune cell infiltration levels. Data retrieved from major public databases, such as TCGA, GEO, GTEx, GSCA, CancerSEA, HPA, and RNAactDrugs, revealed that TUBA1C expression was high in 33 cancer types. Survival analysis revealed that TUBA1C was a poor prognostic factor for 12 tumour types, and mutations, CNVs, and methylation affected the prognosis of some cancer types. Furthermore, TUBA1C was found to be related to immune-related genes, immune cell infiltration, and the immune microenvironment. In addition, the sensitivity of 10 anticancer drugs was associated with high TUBA1C expression. Therefore, TUBA1C may serve as a viable prognostic biomarker for immunotherapy of pan-cancer.

## 1. Introduction

TUBA1C is a member of the TUBA gene family and a component of *α*-microtubules. Microtubules are formed of *α*-tubulin and *β*-tubulin heterodimers, which are organised in the shape of slender filamentous tubes [1]. In addition to being the critical structural components of the eukaryotic cytoskeleton, microtubules perform an integral function in cellular division, formation, mobility, and intracellular transport, after they are organised by globulin/tubulin heterodimers [2, 3]. The biological activity of tubulin is correlated with the onset and progression of cancer, neurodevelopmental disorders, and neurological conditions [4].

*α*-Tubulin is associated with the occurrence of multiple tumours, such as lung, breast, and prostate cancers [5–7]. As a subtype of *α*-tubulin, TUBA1C has remarkably elevated expression in tumour tissues compared with normal tissues [8], and its upregulation indicates a dismal prognosis of pancreatic ductal adenocarcinoma [1]. Furthermore, TUBA1C is involved in the migration and proliferation of hepatocellular carcinoma (HCC) [8] and performs a crucial function in the development of glioma and lung cancer [9, 10]. Given that TUBA1C affects the cell cycle and regulates tumour-infiltrating cells in the tumour microenvironment, its knockdown can suppress the migration of U87and U251 cells (human glioma cells) and induce apoptosis in vitro [11]. TUBA1C may serve as a target for immunotherapy [9, 11]. Although the underlying mechanisms remain unclear, the role of TUBA1C in cancer is attracting increasing attention. However, the function of TUBA1C in the onset and progression of pan-cancer remains unclear.

Although immunotherapy has revolutionised cancer treatment in the past few years, its effectiveness should be improved [12]. In clinical practice, a large proportion of patients does not respond to the available immunotherapy approaches [13]. Therefore, novel immune checkpoints should be investigated. In this study, a comprehensive analysis was performed to assess the role of TUBA1C as an immune checkpoint in the prognosis and treatment of cancer.

To date, studies have mainly focused on the impact of TUBA1C expression on the prognosis of different cancer types; however, the relationship between TUBA1C and mutations, immune-related genes, immune microenvironment, or drug sensitivity has not been examined. In this study, TUBA1C expression was found to be high in several cancer types, and survival analysis revealed that TUBA1C was a poor prognostic factor for >12 cancer types. In addition, the sensitivity of various chemotherapeutic drugs was found to be related to high TUBA1C expression. Therefore, we speculate that TUBA1C can serve as a new site for genetic testing in tumour treatment, thereby improving the prognosis.

## 2. Materials and Methods

### 2.1. Workflow Introduction

**Figure 1:**
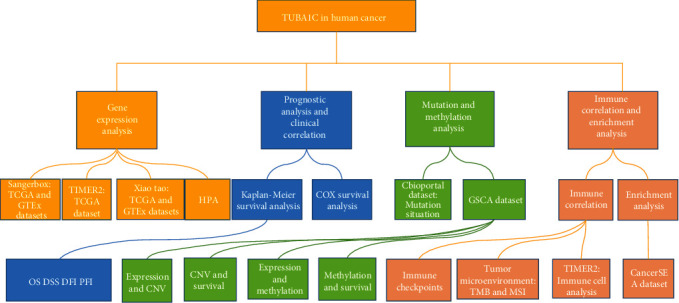
Flowchart of this study.

A flowchart representing the study design has been shown in Figure 1.

### 2.2. Data Acquisition

The UCSC Xena platform (http://xena.ucsc.edu) was used to extract the most recent TUBA1C RNA-sequencing and somatic mutation data and the corresponding clinical data of 33 types of cancer from The Cancer Genome Atlas (TCGA) [14]. The clinical data encompassed progression-free interval (PFI), disease-free interval (DFI), disease-specific survival (DSS), and overall survival (OS). The abbreviations of 33 cancer types are defined in Supplementary Table 1.

### 2.3. TUBA1C mRNA Expression Analysis in Pan-Cancer

The expression data of TUBA1C mRNA in various cancers were extracted from TCGA database. In addition, the Tumor Immune Estimation Resource (version 2) (TIMER2.0; http://timer.cistrome.org/) [15] was used to examine differentially expressed genes (DEGs) and their coexpression status. Subsequently, the differential expression of TUBA1C mRNA was analysed in TCGA tumour types using the “Gene DE” module of TIMER2.0. The Wilcoxon test was used for determining statistical significance. To avoid the impact of the absence of normal samples in some cancer types in TCGA database, a consolidated normalised pan-cancer dataset was downloaded from the UCSC (https://xenabrowser.net/) database [14]. The expression data of TUBA1C were extracted from TCGA and GTEx (PANCANCER, *N* = 19131, *G* = 60499) datasets. Subsequently, the expression data of normal tissues, primary solid tumours and primary tumours, were subjected to log2 (*x* + 0.001) transformation. The R (version 3.6.3) software was used to examine the difference in TUBA1C mRNA expression between tumour and normal samples, and the Wilcoxon signed-rank and rank-sum tests were used to analyse significant differences.

### 2.4. Clinical Prognosis and TUBA1C mRNA Expression

The R (version 3.6.3) software [16] was used to examine the relationship between TUBA1C mRNA expression and the clinical prognosis of various cancer types. Kaplan–Meier curves were generated using the “survminer” and “survival” packages, and Cox regression analysis was performed using the “forestplot”, and “survival” packages. The threshold was established as the median expression value of TUBA1C, and a *p* value of < 0.05 was considered significant.

### 2.5. TUBA1C Protein Expression Profile

The protein expression of TUBA1C was assessed using data obtained from the HPA database (http://www.proteinatlas.org) [17], which is focused on mapping the human proteome in distinct tissues. IHC data from the HPA database and TUBA1C mRNA expression data from TCGA and GTEx databases were compared, and the protein expression of TUBA1C in breast invasive carcinoma (BRCA), colon adenocarcinoma (COAD), lung adenocarcinoma (LUAD), prostate adenocarcinoma (PRAD), and testicular germ cell tumour (TGCT) was compared with that in the corresponding normal tissues.

### 2.6. TUBA1C Mutations in Pan-Cancer

The cBioPortal for Cancer Genomics database [18] is an integrated web resource used for analysing the genomic alterations of specific genes. In this study, the genomic alterations of TUBA1C across 32 cancer types in TCGA were analysed using the OncoPrint tab. In addition, the mutation frequency of TUBA1C was detected; the frequency of alterations in each type of cancer was plotted, and the TUBA1C protein domain and the location of specific mutations were visualised.

### 2.7. GSCA-Based Study of TUBA1C CNVs in Pan-Cancer

Gene Set Cancer Analysis (GSCA; http://bioinfo.life.hust.edu.cn/web/GSCA/) [19] is a cross-over comprehensive cancer analysis database that integrates single-gene, multigene, immune infiltration, mutation, and drug sensitivity analyses of the data of 33 cancer types from TCGA and GDSC. Copy number variations (CNVs) include nonpathogenic CNVs, pathogenic CNVs, and CNVs of unclear clinical value.

The CNV module in GSCA was used to determine the frequency of TUBA1C heterozygosity/purification and amplification/deletion in pan-cancer, Spearman correlation between TUBA1C mRNA expression and CNVs and survival differences between patients with TUBA1C CNVs and wild-type TUBA1C.

### 2.8. GSCA-Based Study of TUBA1C Methylation and m^6^A Modification in Pan-Cancer

The methylation module in GSCA was used to examine differences in TUBA1C methylation between tumour and normal tissue samples, Spearman correlation between TUBA1C methylation and TUBA1C mRNA expression and differences in OS between patients with TUBA1C hypermethylation and hypomethylation.

m^6^A methylation is a common internal modification in mRNA, resulting in the maintenance of mRNA stability. Abnormalities in the enzymes involved in m^6^A modification can contribute to various illnesses such as tumours and neurological diseases [20]. It has been reported that m^6^A-related lncRNA can be used to predict the prognosis of the patients of KIRC [21], therefore, we also explored the relationship between TUBA1C and m^6^A in this study; the correlation between TUBA1C and m^6^A modification-related genes in pan-cancer was examined using the “reshape2” and “RColorBrewer” packages in R.

### 2.9. Relationship between TUBA1C mRNA Expression and the Immune Microenvironment and Immunotherapy of Pan-Cancer Based on TCGA Data

The correlation between TUBA1C and immune-related genes (chemokine receptor-related, immune-suppressive, immune-activation, and MHC genes) in pan-cancer was examined using TCGA data. In addition, the correlation between TUBA1C and mismatch repair- (MMR-) related genes was examined, and Pearson coefficients were calculated. Correlation analysis was performed using the “limma” package in R, and heat maps were generated using the “reshape2” and “RColorBrewer” packages.

Tumour mutational burden (TMB) is calculated as the sum of detected mutations and errors per megabase, which can be used to evaluate the frequency of gene mutations. TMB has a positive correlation with the efficacy of immunotherapy [22]. Microsatellite instability (MSI) is a biological marker that can guide the efficacy of immunotherapy [23]. In this study, the mutation data extracted from TCGA database were used to examine the correlation of TUBA1C mRNA expression with MSI and TMB via Spearman correlation analysis, and the results were visualised using the “fmsb” package.

The correlation between TUBA1C mRNA and the immune microenvironment was examined based on the mesenchymal and immune scores evaluated using the “limma” and “estimate” packages.

### 2.10. Correlation between TUBA1C Expression and Immune Cell Infiltration

**Figure 2:**
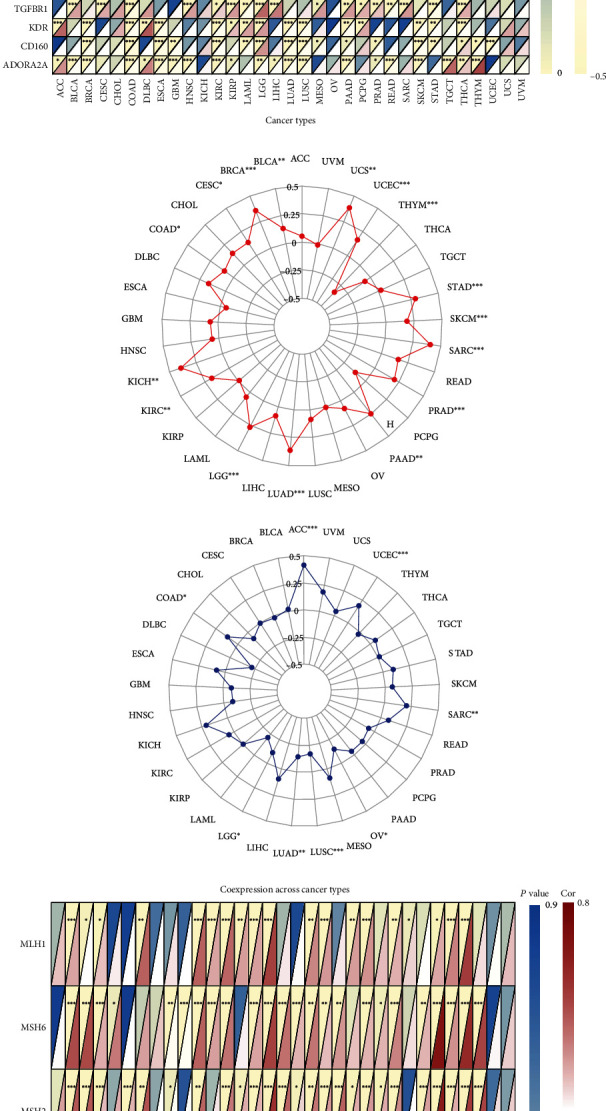
Correlation between TUBA1C mRNA expression and the immune microenvironment. (a) Correlation between TUBA1C and immunosuppressive genes. (b) Correlation between TUBA1C mRNA and TMB. (c) Correlation between TUBA1C mRNA and MSI. (d) Correlation between TUBA1C mRNA and MMR-related genes.

TIMER2.0 was used to examine the correlation between elevated TUBA1C expression and immune cell infiltration to investigate the mechanisms underlying unfavourable prognosis resulting from elevated TUBA1C expression. The relationship among CD4+ T cells, myeloid-derived suppressor cells (MDSCs), and TUBA1C was analysed (Figure 2(a)). In addition, the correlation between TUBA1C expression and the infiltration of CD4+ T cells and MDSCs in pan-cancer was analysed, and their correlation in the four most relevant cancer types is shown in Figures 2(b) and 2(c).

### 2.11. Exploration of Pathways Related to TUBA1C in Pan-Cancer

The functional and expression pathways associated with TUBA1C in pan-cancer were analysed by searching different databases. The CancerSEA database (http://biocc.hrbmu.edu.cn/CancerSEA/) is the first dedicated resource that allows comprehensive analysis of the functioning state of tumour cells at the single-cell level, thus providing details on the varied functions of individual genes in various cancer types at the single-cell level [24], which may help to overcome the limitations of tumour heterogeneity for research.

In this study, the correlation between TUBA1C and the functional status of distinct cancer cells was analysed using CancerSEA, and the effects of TUBA1C on tumour-related pathways were analysed using the “Pathway Activity” module in GSCA.

### 2.12. Analysis of Drug Sensitivity Based on Multiomic Data

Drug sensitivity is key to developing individualised chemotherapy and immunotherapy for cancer. However, owing to the heterogeneity among individuals, the huge difference in drug sensitivity leads to inefficient utilisation of medical resources [25]. Therefore, it is important to study the molecules associated with drug response to optimise drug therapy.

In this study, the RNAactDrug database (http://bio-bigdata.hrbmu.edu.cn/RNAactDrug) [26] was used for drug sensitivity analysis. The GDSC, CellMiner and CCLE data were comprehensively analysed to examine the correlation between the mRNA expression, CNVs, and methylation of TUBA1C and drug sensitivity. Both Pearson and Spearman correlation analyses were used.

## 3. Results

### 3.1. TUBA1C mRNA Expression Patterns in Pan-Cancer

**Figure 3:**
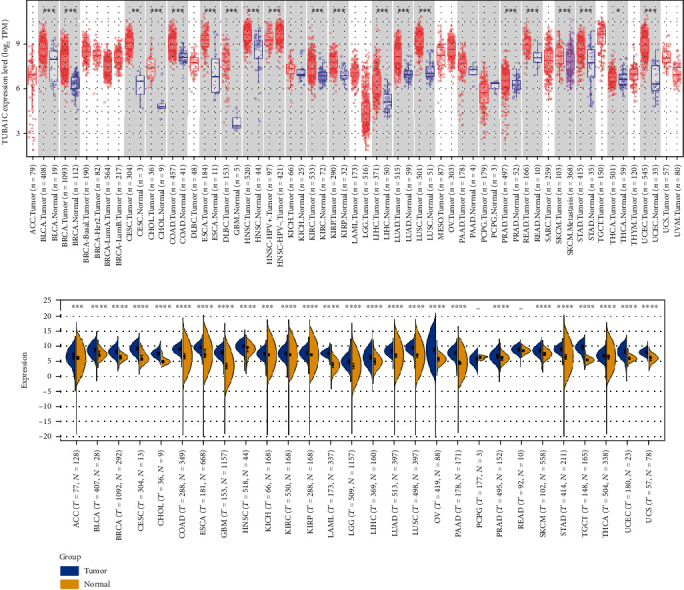
Differential expression of TUBA1C mRNA between cancer and normal tissues. (a) Comparison of TUBA1C mRNA expression between cancer and normal tissues based on TCGA database (red represents tumour tissues, and blue represents normal tissues). (b) Differential expression analysis of TUBA1C mRNA in different cancer types using Sangerbox 3.0 based on TCGA and GTEx data (^∗^*p* < 0.05, ^∗∗^*p* < 0.01, and ^∗∗∗^*p* < 0.001).

The R software (version 3.6.3) was used to examine differences in TUBA1C expression between normal and tumour tissues based on TCGA and GTEx data, and the specific samples and sample characteristics are presented in Supplementary Table 2. Based on TCGA data, TUBA1C expression was upregulated in 27 cancer types (Figure 3(a)), including bladder urothelial carcinoma (BLCA), thyroid carcinoma (THCA), kidney renal papillary cell carcinoma (KIRP), uterine corpus endometrial carcinoma (UCES), skin cutaneous melanoma (SKCM), oesophageal carcinoma (ESCA), pancreatic adenocarcinoma (PAAD), head and neck squamous cell carcinoma (HNSC), liver hepatocellular carcinoma (LIHC), LUAD, glioblastoma multiforme (GBM), kidney chromophobe (KICH), lung squamous cell carcinoma (LUSC), COAD, PRAD, cholangiocarcinoma (CHOL), rectal adenocarcinoma (READ), kidney renal clear cell carcinoma (KIRC), cervical squamous cell carcinoma and endocervical adenocarcinoma (CESC), stomach adenocarcinoma (STAD), and BRCA. Because the proportion of normal tissues in TCGA database was inadequate, normal tissue samples from GTEx were compared with cancer tissue samples from TCGA to analyse differential TUBA1C expression. As shown in Figure 3(b), the mRNA expression of TUBA1C was high in adrenocortical carcinoma (ACC), uterine carcinosarcoma (UCS), testicular germ cell tumour (TGCT), ovarian serous cystadenocarcinoma (OV), brain low-grade glioma (LGG), and acute myeloid leukaemia (LAML). The mRNA expression of TUBA1C was downregulated in only pheochromocytoma and paraganglioma (PCPG).

### 3.2. Correlation between TUBA1C mRNA Expression and Survival in Pan-Cancer

**Figure 4:**
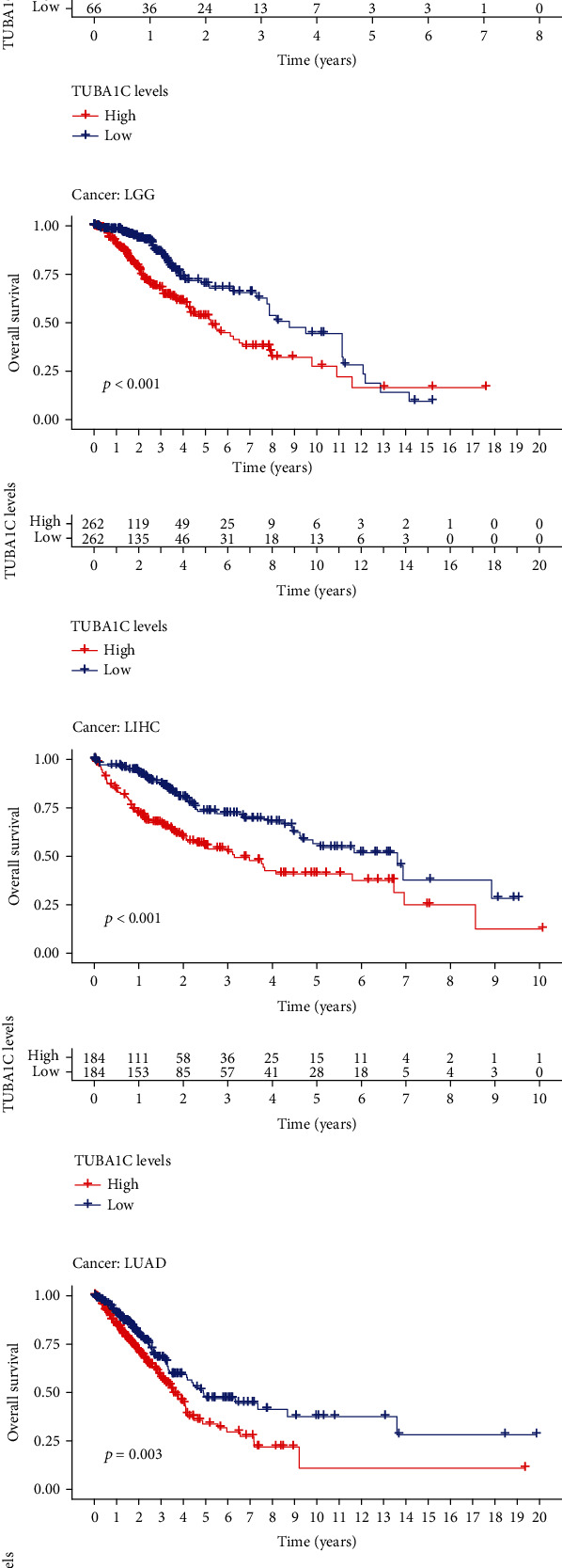
Correlation between TUBA1C mRNA and survival in pan-cancer based on TCGA and GEO data. (a–h): (a) OS curves for BRCA, (b) KIRC, (c) LAML, (d) LGG, (e) LIHC, (f) LUAD, (g) MESO, and (h) SKCM.

**Figure 5:**
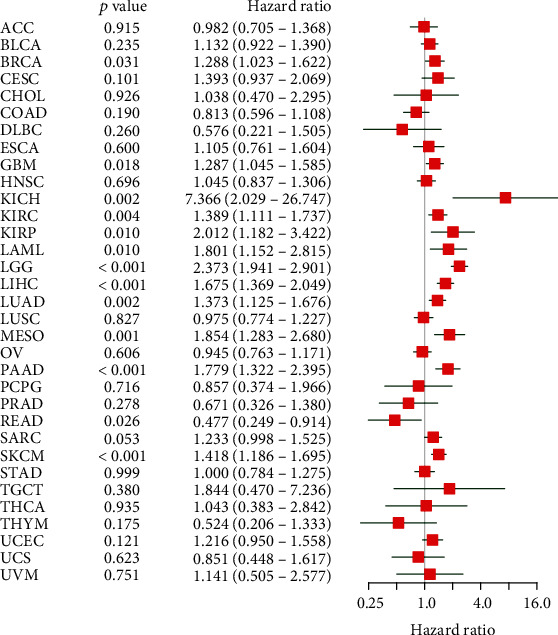
Cox regression analysis was performed to examine the correlation between OS and TUBA1C expression in 33 cancer types in TCGA database. OS: overall survival.

TCGA data were used to examine the role of TUBA1C in predicting PFI, DFI, DSS, and OS in pan-cancer. As shown in Figures 4 and 5, elevated expression of TUBA1C mRNA was correlated with unfavourable OS in KIRC, LAML, LGG, LUAD, mesothelioma (MESO), SKCM, and LIHC. The results of Cox regression analysis revealed that low expression of TUBA1C mRNA was correlated with improved OS in BRCA, GBM, SKCM, KIRC, KIRP, LAML, LGG, LUAD, MESO, LIHC, PAAD, and KICH but with unfavourable OS in rectum adenocarcinoma (READ). Therefore, reduced expression of TUBA1C mRNA is a protective factor for the prognosis of most cancer types but a risk factor for the prognosis of READ.

Similar to the OS curves, DSS curves (Supplementary Figure 1) demonstrated that elevated expression of TUBA1C mRNA was correlated with an unfavourable prognosis of UCEC, SKCM, PAAD, MESO, LIHC, LGG, and KIRC. In addition, the results of Cox regression analysis revealed that reduced expression of TUBA1C mRNA was a protective factor for the DSS of GBM, MESO, LUAD, LIHC, LGG, KIRP, KIRC, PAAD, KICH, and SKCM but a risk factor for the prognosis of PRAD.

Furthermore, DFI curves (Supplementary Figure 2) revealed that high expression of TUBA1C mRNA was correlated with an unfavourable prognosis of PAAD, LUAD, and sarcoma (SARC). The results of Cox regression analysis revealed that low expression of TUBA1C mRNA was a protective factor for the DFI of SARC, PAAD, LUAD, and LGG.

PFI curves (Supplementary Figure 3) revealed that high expression of TUBA1C mRNA was correlated with an unfavourable prognosis of SARC, PAAD, MESO, LUAD, LGG, KIRC, KICH, GBM, and ACC, whereas low expression of TUBA1C mRNA was correlated with an unfavourable prognosis of READ. In addition, the results of Cox regression analysis revealed that low expression of TUBA1C mRNA was a protective factor for the PFI of GBM, UCEC, SKCM, SARC, PAAD, MESO, LUAD, LIHC, LGG, KIRC, and KICH but was a risk factor for the PFI of COAD.

### 3.3. TUBA1C Protein Expression Profile Based on HPA and TCGA Data

**Figure 6:**
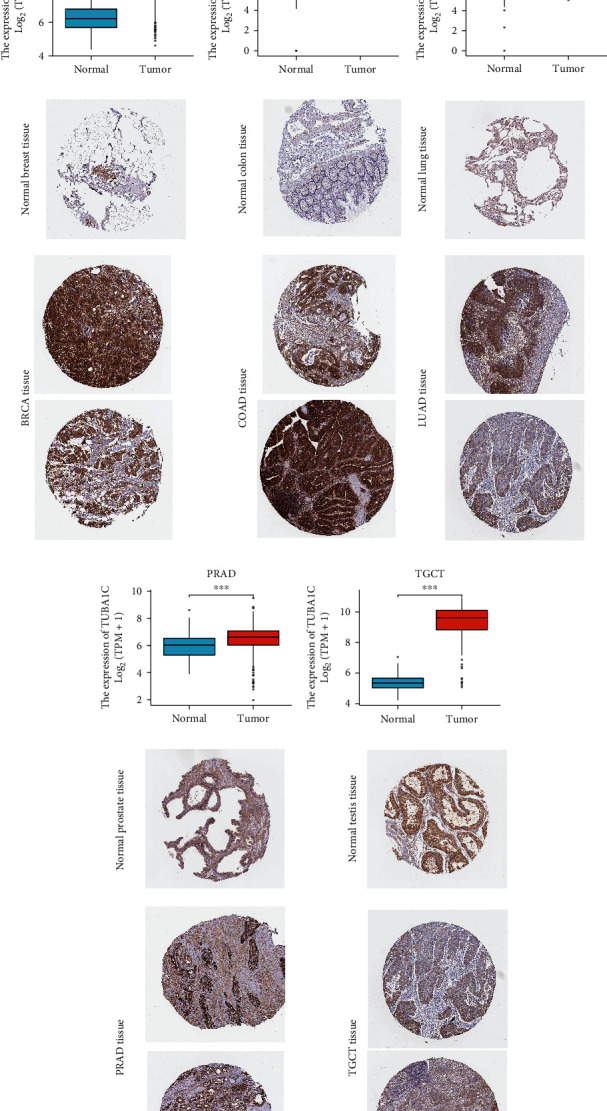
TUBA1C protein expression in tumour and normal tissues based on TCGA and GTEx data. (a) TUBA1C is mainly expressed in the cytoplasm and cell membrane. (b–f) The protein expression profile of TUBA1C in tumour and normal tissues.

IHC data extracted from the HPA database and TUBA1C mRNA expression data extracted from TCGA and GTEX were compared to analyse TUBA1C expression at the protein level. As shown in Figure 6(a), TUBA1C, like microtubules, is mainly expressed in the cytoplasm and cell membrane but not in the nucleus. As shown in Figures 6(b)–6(f), analysis of data retrieved from the two databases yielded the same results. The intensity of TUBA1C staining was low in healthy colon and lung tissues but high in malignant tissues. The staining intensity was moderate in healthy breast tissues but high in tumour tissues. In addition, the staining intensity was moderate in healthy prostate tissues but high in malignant tissues. However, both healthy and tumour testicular tissues had moderate staining intensity. This finding was different from that obtained via differential expression analysis of TUBA1C mRNA, which may be related to the small number of IHC staining samples in the HPA database.

### 3.4. TUBA1C Mutation Analysis in Pan-Cancer Using cBioPortal

**Figure 7:**
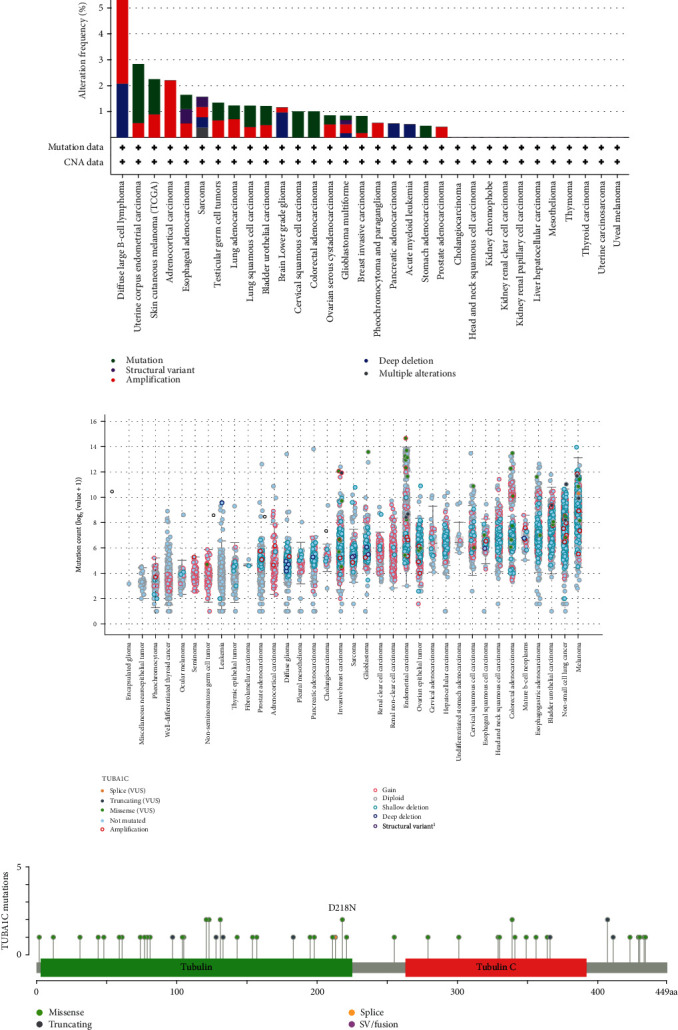
TUBA1C mutation analysis in pan-cancer using cBioPortal. (a) The mutation frequency of TUBA1C in different cancers. (b) The mutation count of TUBA1C in different cancers. (c) The mutation sites of TUBA1C.

The mutation frequency of TUBA1C in TCGA cohort (10,105 samples spanning 32 cancer types) was examined using the cBioPortal database. As shown in Figures 7(a) and 7(b), diffuse large B-cell lymphoma had the highest mutation levels (>6%), the highest frequency of amplification alterations (4.17%) and the highest frequency of deep deletion alterations (2.08%) across the 32 cancer types. In addition, as shown in Figure 7(c), TUBA1C has 58 mutation sites (including 44 missense mutations, 8 truncation mutations, 1 splice mutation, and 6 fusion mutations), which are located between 0 and 449 amino acids. The mutation frequency of P364T was the highest.

### 3.5. GSCA-Based Study of TUBA1C CNVs in Pan-Cancer

**Figure 8:**
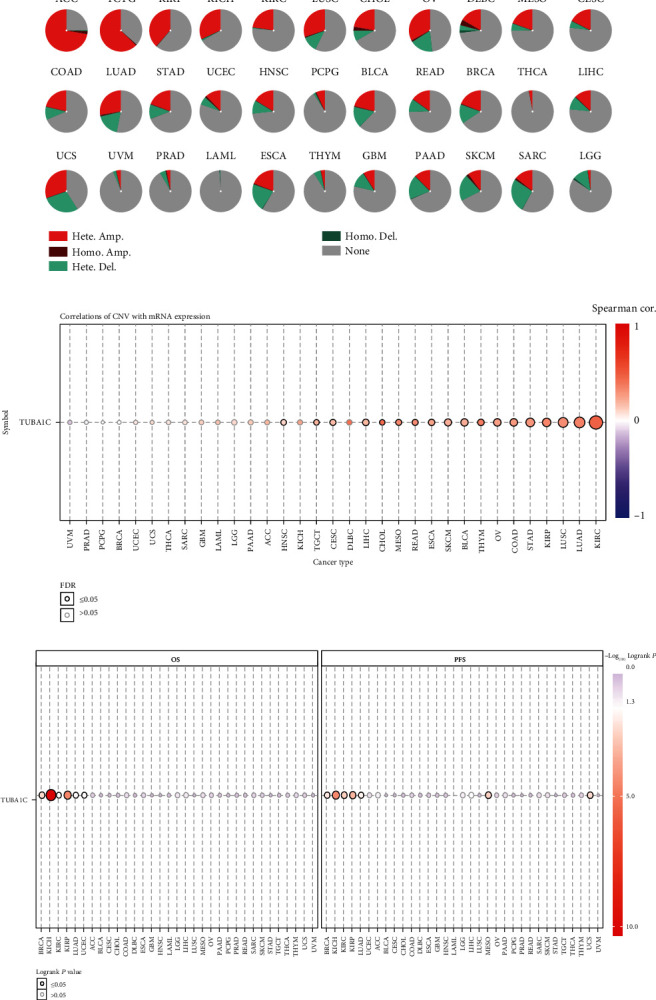
CNV analysis of TUBA1C in pan-cancer using GSCA. (a) The count of deletion, amplification, heterozygous, and homozygous CNVs for TUBA1C in different tumours. (b) Correlation between the CNVs and mRNA expression of TUBA1C in different tumours. (c) Differences in survival between patients with wild-type TUBA1C and TUBA1C CNVs in pan-cancer.

The CNVs of TUBA1C in 33 cancer types were examined using GSCA. As shown in Figure 8(a), the rate of heterozygous amplification was higher in ACC and TCTG (>50%), whereas the rate of heterozygous deletion was <20% in most cancer types but >20% in UCS, SARC, ESCA, and SKCM. Supplementary Table 3 provides detailed information on CNVs observed in each cancer type. A significant correlation was observed between the CNVs and mRNA expression of TUBA1C in 18 cancer types, (Figure 8(b) and Supplementary Table 4). Furthermore, the prognostic significance of TUBA1C CNVs in pan-cancer was examined (Figure 8(c)). The results showed that compared with TUBA1C variant with copy number deletion/amplification, wild-type TUBA1C remarkably improved OS in KIRC, LUAD, KICH, BRCA, UCEC, and KIRP and PFS in BRCA, KICH, KIRC, KIRP, LUAD, MESO, and UCS. In addition, TUBA1C variant with copy number deletion resulted in better PFS and OS in BRCA than TUBA1C variant with copy number amplification. Supplementary Figure 4 depicts a comprehensive representation of the survival curves.

### 3.6. GSCA-Based Study of TUBA1C Methylation and m^6^A-Related Genes in Pan-Cancer

**Figure 9:**
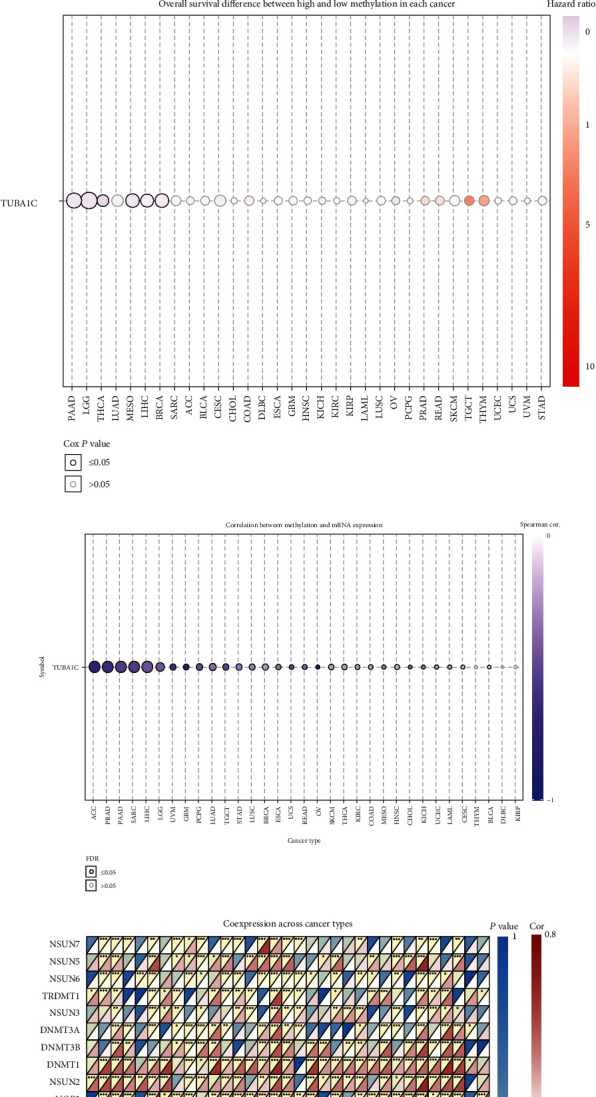
GSCA-based study on TUBA1C methylation and m^6^A-related genes in pan-cancer. (a) Differences in TUBA1C methylation among different cancer types. (b) Differences in overall survival between high- and low-methylation samples in each cancer type. (c) Correlation between methylation and mRNA expression in different cancer types. (d) Correlation between m^6^A-related genes and TUBA1C mRNA in different cancer types.

GSCA was used to examine TUBA1C methylation in pan-cancer. TUBA1C methylation in COAD, PRAD, PAAD, THCA, HNSC, ESCA, LIHC, BRCA, UCES, KIRC, LUAD, KIRP, and LUSC was compared with that in normal tissues (Figure 9(a)) and was considerably associated with TUBA1C mRNA expression in most cancer types (Figure 9(c)). In addition, TUBA1C hypermethylation was correlated with better OS in PAAD, LGG, THCA, MESO, LIHC, and BRCA (Figure 9(b)). Detailed information is provided in Supplementary Table 5.

Furthermore, a positive correlation was observed between m^6^A-related genes and TUBA1C mRNA (Figure 9(d)) in most cancer types, especially in LIHC and TGCT. In these two cancer types, TUBA1C showed a positive correlation with all m^6^A-related genes.

### 3.7. Correlation between TUBA1C mRNA Expression and the Immune Microenvironment and Immunotherapy of Pan-Cancer

**Figure 10:**
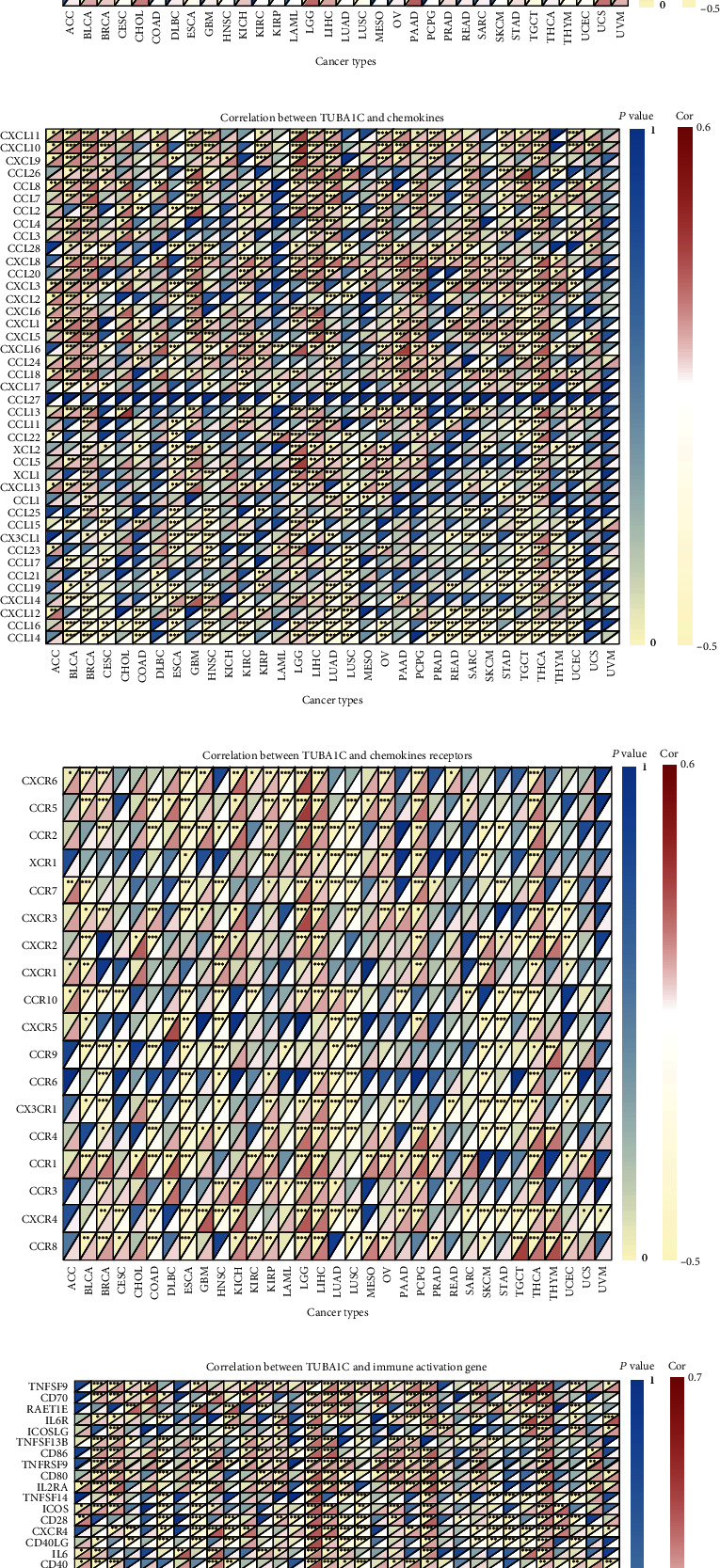
Correlation between TUBA1C mRNA expression and the immune microenvironment. (a) Correlation between TUBA1C and MHC-related genes. (b) Correlation between TUBA1C and chemokines. (c) Correlation between TUBA1C and chemokine receptors. (d) Correlation between TUBA1C and immune activation genes.

TCGA data were used to examine the correlation between TUBA1C mRNA and MHC-related genes in pan-cancer. As shown in Figure 2(a), TUBA1C was positively correlated with all MHC-related genes in LGG and LIHC and with the majority of MHC-related genes in THCA, BLCA, and BRCA. In particular, the TAP1 gene was positively correlated with TUBA1C in almost all cancer types, indicating that TAP1 can serve as a promising immunotherapy target. As shown in Figure 10(c), TUBA1C was positively correlated with CD276, PVR, and MICB in most cancer types. As shown in Figures 10(a), 10(b), and 10(d), TUBA1C was positively correlated with some immune-related genes in most cancer types. In particular, it was positively correlated with almost all immunosuppressive, chemotactic and chemotactic receptor genes in LGG, LIHC, and THCA, suggesting that TUBA1C influences the prognosis of these three cancer types through immune-related genes.

Furthermore, as shown in Figure 2(d), TUBA1C mRNA had a positive correlation with MMR-related genes (MLH1, MSH6, MSH2, PMS2, and EPCAM) in most cancer types, with the strongest correlation observed in LIHC, PRAD, and TGCT.

As shown in Figure 2(b), TUBA1C mRNA was significantly positively correlated with TMB in BLCA, BRCA, CESC, KICH, PAAD, LGG, PRAD, KIRC, SARC, COAD, SKCM, STAD, LUAD, UCEC, and UCS, with the strongest correlation observed in UCS, SARC, KICH, and BRCA. However, TUBA1C mRNA and TMB were negatively correlated in THYM. Furthermore, TUBA1C mRNA and MSI had a positive correlation in ACC, UCEC, SARC, and COAD but a negative correlation in OV, LUSC, LUAD, and LGG (Figure 2(c)).

The cytolytic activity (CYT) score is an immune activity score obtained by calculating the mRNA expression of GZMA and PRF1 genes (CYT score = √GZMA × PRF1). In recent years, the CYT score has been used to predict the prognosis of some tumours. Low CYT scores represent weak antitumour immunity in GC, resulting in a poor prognosis [27] and are also associated with poor outcomes in HCC [28].

In this study, CYT scores and TUBA1C were positively correlated in most cancer types, especially in LGG, OV, GBM, and BLCA (Supplementary Figure 5), which indicates that TUBA1C affects the prognosis of patients by affecting the activity of immune cells and the immune microenvironment.

**Figure 11:**
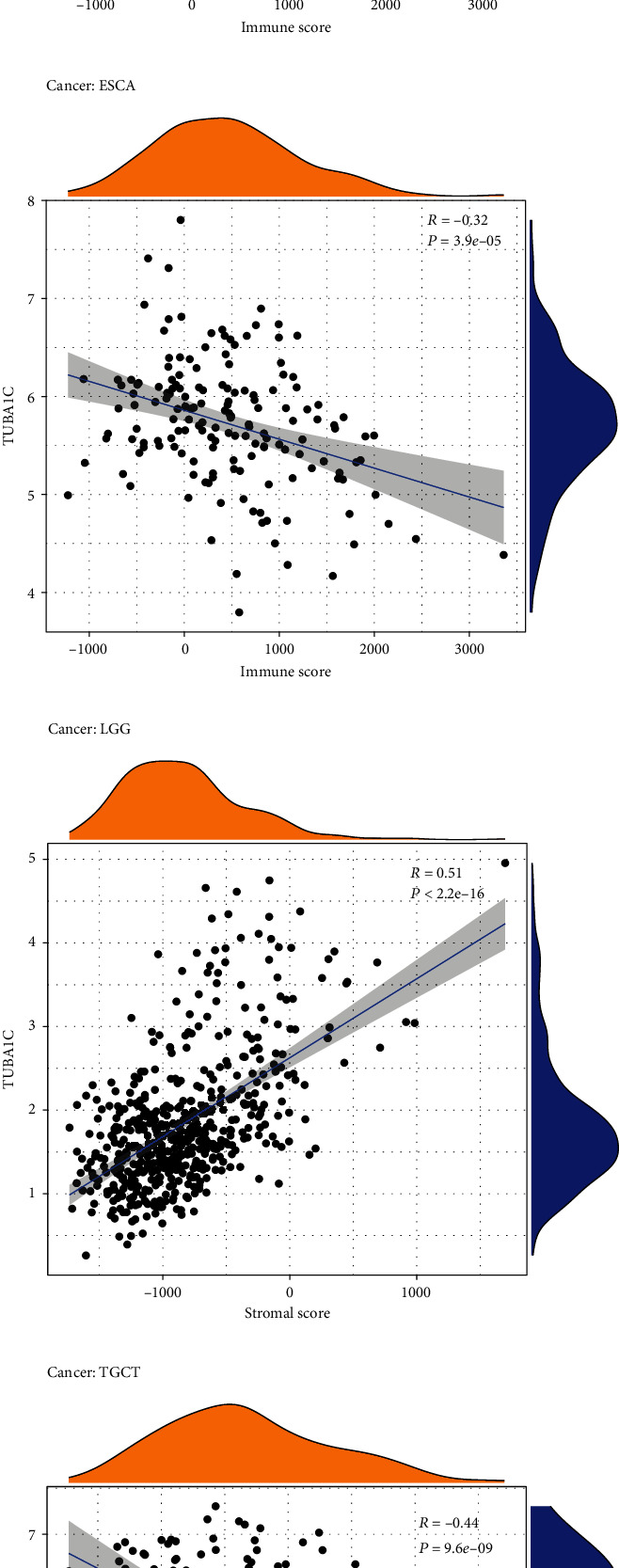
The relationship between TUBA1C mRNA and the immunological microenvironment. (a–b) Four cancer types with the highest immune scores (LGG, GBM, THCA, and ESCA). (e–h) Four cancer types with the highest interstitial scores (LGG, GBM, TGCT, and PCPG).

In addition, the relationship between TUBA1C mRNA and the immunological microenvironment was investigated. The four cancer types with the highest immune scores (LGG, GBM, THCA, and ESCA), and the four cancer types with the highest interstitial scores (LGG, GBM, TGCT, and PCPG) are shown in Figures 11(a)–11(h) (details are shown in Supplementary Figure 6). A negative correlation was observed between TUBA1C mRNA expression and immune scores in ESCA and TGCT.

### 3.8. Correlation between TUBA1C Expression and Immune Cell Infiltration

**Figure 12:**
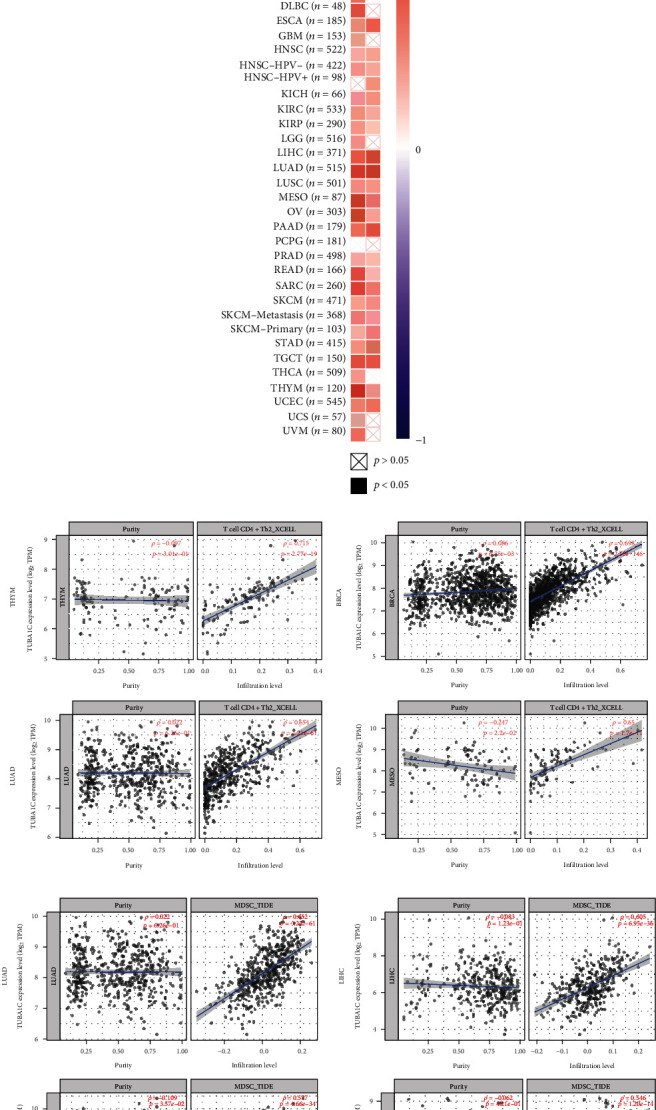
Correlation between TUBA1C and the infiltration of CD4+ T cells and MDSCs. MDSCs: myeloid-derived suppressor cells. (a) TUBA1C was positively correlated with the infiltration of CD4+ T cells and MDSCs in different cancer types. (b) TUBA1C expression had the strongest correlation with the infiltrating levels of CD4+ T cells in THYM, BRCA, LUAD, and MESO. (c) TUBA1C expression had the strongest correlation with the infiltrating levels of MDSCs in LUAD, LIHC, BLCA, and PAAD.

TIMER2.0 was used to visualise the relationship between TUBA1C expression and immune infiltration levels in different cancer types. As shown in Figure 12(a), TUBA1C was positively correlated with the infiltration of CD4+ T cells and MDSCs in different cancer types, and the specific correlation coefficients are shown in Table 1. As shown in Figure 12(b), TUBA1C had the strongest correlation with the infiltrating levels of CD4+ T cells in THYM, BRCA, LUAD, and MESO, with the correlation coefficients of 0.715, 0.698, 0.654, and 0.65, respectively. In addition, TUBA1C had the strongest correlation with the infiltrating levels of MDSCs in LUAD, LIHC, BLCA, and PAAD, with the correlation coefficients of 0.652, 0.605, 0.577, and 0.546, respectively. These results indicate that TUBA1C may affect the prognosis by affecting the infiltration of CD4+ T cells and MDSCs. MDSCs are a heterogeneous group of bone marrow-derived cells that can substantially suppress immune cell responses [29], which may be a novel target for immunotherapy.

### 3.9. Pathway Analysis of TUBA1C in Pan-Cancer

**Figure 13:**
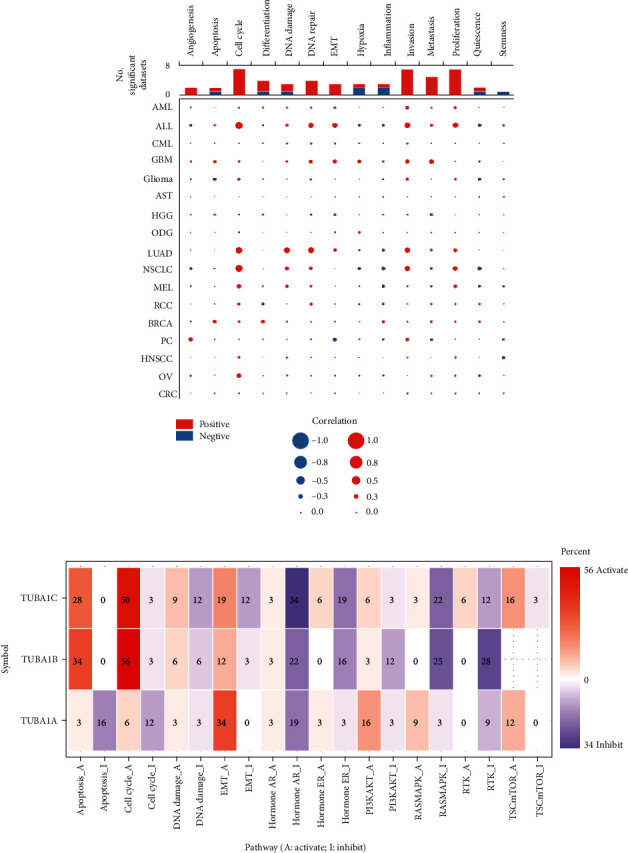
Pathway analysis of TUBA1C in pan-cancer based on GSCA and CancerSEA databases. (a) TUBA1C is associated with various functional states in most cancers. (b) Functions of the TUBA family in pan-cancer-related pathways.

The CancerSEA database was used to determine the functional state of TUBA1C in different cancer types at the single-cell level. As shown in Figure 13(a), TUBA1C was correlated with various functional states of TUBA1C in most cancer types. TUBA1C was found to have a positive correlation with cell cycle, DNA repair, EMT, invasion and proliferation in ALL and with cell cycle, DNA damage, DNA repair, and invasion and proliferation in NSCLC and LUAD.

Furthermore, GSCA was used to examine the role of the TUBA family in tumour-related pathways to distinguish the functions of TUBA1C from those of other members of the TUBA family (TUBA1A and TUBA1B).

As shown in Figure 13(b), TUBA1C and TUBA1B can activate apoptosis, promote the cell cycle, and inhibit AR and RAS/MAPK, whereas TUBA1A inhibits the cell cycle and apoptosis. These results indicate that TUBA1C and TUBA1B play a similar role in pan-cancer and are different from TUBA1A.

### 3.10. Drug Sensitivity Analysis

The RNAactDrug database was used to assess the relationship between the mRNA expression, CNVs, and methylation of TUBA1C and anticancer drug sensitivity. As shown in Table 2, the anticancer drug sensitivity of tivozanib, ruxolitinib, OSI-027, GSK690693, linifanib, AT-7519, CUDC-101, tamoxifen, CP466722, and belinostat was positively correlated with TUBA1C mRNA expression; that of daporinad, ispinesib mesylate, navitoclax, CX-5461, SNX-2112, TL-1-85, 5-fluorouracil, vismodegib, PHA-793887, and QL-XL-92 was positively correlated with TUBA1C CNVs and that of PHA-793887, GSK690693, BMS-345541, TAK-715, bleomycin (50 *μ*M), QL-X-138, methotrexate, OSI-930, and tivozanib was negatively correlated with TUBA1C mRNA methylation.

## 4. Discussion

To the best of our knowledge, this study is the first to report multiomic analysis of TUBA1C in pan-cancer. Specifically, the expression of TUBA1C mRNA in various cancer types was analysed and verified using data extracted from TCGA and GTEx databases. Furthermore, the relationship between TUBA1C expression and survival was analysed using TCGA database, and proteomic analysis was conducted using the HPA database. The prognostic significance of the CNVs, methylation, and m^6^A modification of TUBA1C was investigated based on data extracted from several databases, and pathways associated with TUBA1C were assessed using CancerSEA and TCGA databases. In addition, the correlation of TUBA1C with the immunological microenvironment, immunotherapy, and targeted therapy was examined at the multiomic level. The results suggest that TUBA1C is a promising prognostic biomarker for immunotherapy of pan-cancer.

Previous studies have shown that elevated expression of TUBA1C leads to an unfavourable prognosis in pancreatic ductal adenocarcinoma [1]. In addition, TUBA1C is associated with a poor prognosis in HCC, glioma, and lung adenocarcinoma [8–11], which is consistent with the results of the present study. In this study, TUBAC was found to be overexpressed in most cancer types, which was verified via proteomic analysis (high expression of TUBA1C protein in COAD, LUAD, BRCA, and PRAD). Furthermore, low expression of TUBA1C was found to be a protective factor for OS, PFI, and DFI in most cancer types but was found to be a risk factor for PRAD and COAD. Therefore, although the role of TUBA1C may be heterogeneous in different tumours, TUBA1C mRNA expression can be used to predict the prognosis of some tumours.

Genomic instability and mutations are common features of tumours. Genomic instability is caused by mutations in DNA repair genes and drives cancer development [30]. Therefore, we examined whether TUBA1C has mutations, and whether these mutations affect the prognosis of tumours. The cBioPortal database was used to evaluate the mutation frequency of TUBA1C and its specific mutation sites in 32 cancer types. TUBA1C mutations were found in 21 of the 32 cancer types, and diffuse large B-cell lymphoma had relatively higher mutation levels (>6%). Furthermore, TUBA1C had 58 mutation sites (including 44 missense mutations, 8 truncating mutations, 1 splicing mutation, and 6 fusion mutations) located between 0 and 449 amino acids, and the mutation frequency of P364T was the highest. Gene mutations are often associated with a poor prognosis, and CNVs are one of the major forms of genetic alterations in cancer, with different patterns of CNVs across cancer types [31]. In this study, the correlation between the CNVs and mRNA expression of TUBA1C was significant. Compared with TUBA1C variant with copy number amplification/deletion, wild-type TUBA1C was associated with remarkably improved PFS and OS in BRCA, KICH, KIRC, KIRP, and LUAD, which indicates the prognostic value of TUBA1C CNVs.

In recent years, methylation has received increasing attention. It is an important modification in proteins and nucleic acids, is closely related to many diseases such as cancer, ageing, and senile dementia, and is an important part of epigenetics [32]. In this study, TUBA1C methylation was found to be associated with TUBA1C mRNA expression in a majority of tumours, and its hypermethylation resulted in better OS in PAAD, LGG, THCA, MESO, LIHC, and BRCA, which indicates the prognostic value of TUBA1C methylation.

In addition to DNA methylation, internal RNA modifications are also important in tumour development, especially m^6^A modification, which is the most prevalent methylation modification in eukaryotic RNA, accounting for >80% of all RNA methylation [33]. In this study, TUBA1C and m^6^A-related genes were positively correlated in most cancer types. In particular, TUBA1C was positively correlated with all m^6^A-related genes in LIHC and TGCT, suggesting that TUBA1C affects cancer development by affecting m^6^A modification in LIHC and TGCT.

Immune checkpoint inhibitors, which are antagonistic antibodies, have emerged as an innovative treatment option for cancer in the last few years, which reshape the paradigm of oncological treatment [14]. However, a large proportion of patients does not benefit from immunotherapy; therefore, screening for dominant groups is important [34]. In this study, the correlation between TUBA1C and immune-associated genes (chemotactic receptor, immunosuppressive, immune activation, chemotactic, and MHC-related genes), MMR-related genes (MLH1, MSH6, MSH2, PMS2, and EPCAM), immune scores, and interstitial scores was analysed. The results showed that TUBA1C had a positive correlation with most MHC-related genes in THCA, BLCA, and BRCA, and the TAP1 gene was positively correlated with TUBA1C in almost all cancer types, suggesting that TAP1 is a potential immunotherapeutic target. Furthermore, TUBA1C exhibited a positive correlation with almost all immunosuppressive, chemotactic, and chemotactic receptor genes in LGG, LIHC, and THCA, suggesting that TUBA1C influences tumour development through immune-related genes in these cancers. Furthermore, TUBA1C mRNA had a positive correlation with MMR-related genes (MLH1, MSH6, PMS2, MSH2, and EPCAM) in most cancer types and with TMB in UCS, UCEC, STAD, SKCM, SARC, PRAD, PAAD, LUAD, LGG, KIRC, KICH, COAD, CESC, BRCA, and BLCA. In addition, TUBA1C mRNA and MSI were positively correlated in ACC, UCEC, SARC, and COAD. These results indicate that TUBA1C is a potential indicator for screening dominant populations that can benefit from immunotherapy.

Previous studies have demonstrated that elevated expression of TUBA1C may contribute to an unfavourable prognosis in LGG and LUAD by increasing the infiltration of dendritic cells, neutrophils, macrophages, CD4+ T cells, CD8 T+ cells, and B cells [9, 10]. In this study, the relationship between TUBA1C and the immune microenvironment of pan-cancer was examined by analysing the correlation between TUBA1C and immune cell infiltration. The results demonstrated a positive correlation between TUBA1C and the infiltration of CD4+ T cells and MDSCs in most cancer types. MDSCs are considered the “queen bee” in the tumour microenvironment, which can protect tumours from the immune system of patients, thereby making tumours resistant to immunotherapy and promoting tumour growth [35]. In this study, TUBA1C was strongly correlated with MDSCs in pan-cancer, indicating that MDSCs may be used to develop novel immunotherapy strategies in the future.

As a component of *α*-tubulin, TUBA1C plays a role in several processes, including cell division, formation, and movement. Studies have reported that knockout of TUBA1C in vitro can significantly inhibit the proliferation of pancreatic cancer cells and promote apoptosis [1], and TUBA1C expression may alter the prognosis of HCC through cell cycle signalling [8]. In this study, the CancerSEA and GSCA databases were used to examine the mechanism of action of TUBA1C in pan-cancer. The results showed that TUBA1C activated apoptosis and promoted the cell cycle in pan-cancer, which is consistent with the findings of previous studies. However, the specific mechanism of action of TUBA1C warrants further investigation.

Furthermore, the relationship between TUBA1C mRNA and anticancer drug sensitivity was examined. To the best of our knowledge, the correlation between TUBA1C and the sensitivity of specific anticancer drugs has not yet been reported; however, this study showed that the sensitivity of some anticancer drugs was correlated with the mRNA expression and CNVs of TUBA1C. Most of these drugs are kinase inhibitors and also include anticancer drugs such as ispinesib mesylate and PHA-793887 that inhibit the cell cycle. As mentioned earlier, cell cycle regulation is one of the important pathways associated with TUBA1C in pan-cancer. Therefore, we speculate that TUBA1C is the target of some anticancer drugs or a promising biomarker for predicting drug sensitivity. However, the study of TUBA1C in tumour therapy is subjected to certain limitations, thus necessitating additional investigation.

## 5. Conclusions

In this study, we reported for the first time the multiomics analysis of TUBA1C in pan-cancer, and we found that the high expression of TUBA1C is associated with poor prognosis in most tumour patients, and its expression level and mutation also affect the prognosis of patients, and whether TUBA1C is highly expressed can be used to search the dominant groups for immunotherapy. Overall, we believe that this study can provide a new idea for future cancer treatment. However, like all other retrospective studies, our study also has certain limitations. First, the data in the existing databases are not comprehensive, and second, limited to laboratory conditions, we have not been able to carry out experimental verification and clinical trials. Therefore, future studies can further validate the role of TUBA1C in tumours through clinical trials or prospective studies.

## Figures and Tables

**Table 1:** Correlation between TUBA1C and the infiltration of CD4+ T cells and MDSCs in different cancer types. MDSCs: myeloid-derived suppressor cells (^∗^*p* < 0.05, ^∗∗^*p* < 0.01, and ^∗∗∗^*p* < 0.001).

	MDSC		T cell CD4+ Th2	
	Rho	Adj.p	Rho	Adj.p
ACC (*n* = 79)	0.44	^∗∗∗^	0.33	^∗^
BLCA (*n* = 408)	0.58	^∗∗∗^	0.62	^∗∗∗^
BRCA (*n* = 1100)	0.46	^∗∗∗^	0.70	^∗∗∗^
BRCA-basal (*n* = 191)	0.22	^∗∗^	0.36	^∗∗∗^
BRCA-Her2 (*n* = 82)	0.29	^∗^	0.45	^∗∗∗^
BRCA-LumA (*n* = 568)	0.29	^∗∗∗^	0.48	^∗∗∗^
BRCA-LumB (*n* = 219)	0.39	^∗∗∗^	0.43	^∗∗∗^
CESC (*n* = 306)	0.21	^∗∗^	0.22	^∗∗^
CHOL (*n* = 36)	0.16	0.49	0.16	0.49
COAD (*n* = 458)	0.04	0.56	0.44	^∗∗^
DLBC (*n* = 48)	0.20	0.32	0.55	^∗∗^
ESCA (*n* = 185)	0.49	^∗∗∗^	0.33	^∗∗∗^
GBM (*n* = 153)	0.16	0.14	0.28	^∗∗^
HNSC (*n* = 522)	0.25	^∗∗∗^	0.22	^∗∗∗^
HNSC-HPV- (*n* = 422)	0.23	^∗∗∗^	0.29	^∗∗∗^
HNSC-HPV+ (*n* = 98)	0.31	^∗^	0.08	0.58
KICH (*n* = 66)	0.29	^∗^	0.26	0.08
KIRC (*n* = 533)	0.23	^∗∗∗^	0.31	^∗∗∗^
KIRP (*n* = 290)	0.15	^∗^	0.28	^∗∗∗^
LGG (*n* = 516)	0.07	0.27	0.29	^∗∗∗^
LIHC (*n* = 371)	0.61	^∗∗∗^	0.51	^∗∗∗^
LUAD (*n* = 515)	0.65	^∗∗∗^	0.65	^∗∗∗^
LUSC (*n* = 501)	0.28	^∗∗∗^	0.30	^∗∗∗^
MESO (*n* = 87)	0.42	^∗∗∗^	0.65	^∗∗∗^
OV (*n* = 303)	0.23	^∗∗∗^	0.62	^∗∗∗^
PAAD (*n* = 179)	0.55	^∗∗∗^	0.43	^∗∗∗^
PCPG (*n* = 181)	-0.12	0.22	0.07	0.53
PRAD (*n* = 498)	0.17	^∗∗^	0.21	^∗∗∗^
READ (*n* = 166)	0.18	0.09	0.58	^∗∗∗^
SARC (*n* = 260)	0.38	^∗∗∗^	0.60	^∗∗∗^
SKCM (*n* = 471)	0.31	^∗∗∗^	0.25	^∗∗∗^
SKCM-metastasis (*n* = 368)	0.27	^∗∗∗^	0.35	^∗∗∗^
SKCM-primary (*n* = 103)	0.34	^∗∗^	0.23	0.06
STAD (*n* = 415)	0.46	^∗∗∗^	0.30	^∗∗∗^
TGCT (*n* = 150)	0.53	^∗∗∗^	0.55	^∗∗∗^
THCA (*n* = 509)	-0.03	0.66	0.27	^∗∗∗^
THYM (*n* = 120)	0.30	^∗∗∗^	0.72	^∗∗∗^
UCEC (*n* = 545)	0.41	^∗∗∗^	0.36	^∗∗^
UCS (*n* = 57)	0.13	0.50	0.28	0.10
UVM (*n* = 80)	0.08	0.62	0.42	^∗∗∗^

**Table 2:** Drug sensitivity analysis: correlation between the mRNA expression, CNVs, and methylation of TUBA1C and anticancer drug sensitivity.

Drug	RNA type	RNA molecule	Omics	Source	Pearson. Cor	Pearson. FDR	Spearman. Cor	Spearman. FDR
Tivozanib	mRNA	TUBA1C	Expression	GDSC	0.1181432	0.000714333	0.244229939	2.95*E* − 13
Ruxolitinib	mRNA	TUBA1C	Expression	GDSC	0.1128282	0.001262985	0.244182065	3.09*E* − 13
OSI-027	mRNA	TUBA1C	Expression	GDSC	0.1173959	0.000866345	0.235973875	4.01*E* − 12
GSK690693	mRNA	TUBA1C	Expression	GDSC	0.1181802	0.000692556	0.230212195	1.04*E* − 11
Linifanib	mRNA	TUBA1C	Expression	GDSC	0.1005532	0.006125521	0.21276724	1.18*E* − 09
AT-7519	mRNA	TUBA1C	Expression	GDSC	0.10040892	0.004489092	0.21047621	6.34*E* − 10
CUDC-101	mRNA	TUBA1C	Expression	GDSC	0.12304633	0.000600801	0.19774505	1.13*E* − 08
Tamoxifen	mRNA	TUBA1C	Expression	GDSC	0.1136837	0.001482619	0.197685562	1.21*E* − 08
CP466722	mRNA	TUBA1C	Expression	GDSC	0.12349821	0.000412708	0.197639711	6.71*E* − 09
Belinostat	mRNA	TUBA1C	Expression	GDSC	0.1193443	0.000952147	0.195656114	1.38*E* − 08
Daporinad	mRNA	TUBA1C	CNV	GDSC	0.11523845	0.000816374	0.13490644	7.32*E* − 05
Ispinesib Mesylate	mRNA	TUBA1C	CNV	GDSC	0.119002	0.000706378	0.128803562	0.000159665
Navitoclax	mRNA	TUBA1C	CNV	GDSC	0.1116253	0.001206781	0.120060079	0.000454901
CX-5461	mRNA	TUBA1C	CNV	GDSC	0.0999689	0.003371957	0.108937723	0.001351591
SNX-2112	mRNA	TUBA1C	CNV	GDSC	0.1018199	0.004809037	0.107863783	0.001966635
TL-1-85	mRNA	TUBA1C	CNV	GDSC	0.1223173	0.000392341	0.107727422	0.001719903
5-fluorouracil	mRNA	TUBA1C	CNV	GDSC	0.11008902	0.001395387	0.105981393	0.002212679
Vismodegib	mRNA	TUBA1C	CNV	GDSC	0.1035804	0.00371599	0.101283742	0.003856671
PHA-793887	mRNA	TUBA1C	CNV	GDSC	0.1057712	0.003508679	0.09876329	0.00622636
QL-XI-92	mRNA	TUBA1C	CNV	GDSC	0.1047695	0.0028476	0.09828511	0.005003631
PHA-793887	mRNA	TUBA1C	Methylation	GDSC	-0.101829	0.004264568	-0.309603131	1.38*E* − 19
WZ3105	mRNA	TUBA1C	Methylation	GDSC	—	—	-0.30266043	1.96*E* − 18
GSK690693	mRNA	TUBA1C	Methylation	GDSC	-0.1113654	0.001879128	-0.298473273	5.90*E* − 18
BMS-345541	mRNA	TUBA1C	Methylation	GDSC	-0.12062933	0.000764463	-0.287012122	8.24*E* − 17
TAK-715	mRNA	TUBA1C	Methylation	GDSC	-0.1165397	0.001056131	-0.280318734	4.96*E* − 16
Bleomycin (50 *μ*M)	mRNA	TUBA1C	Methylation	GDSC	0.1162832	0.002971015	0.272904591	1.28*E* − 14
QL-X-138	mRNA	TUBA1C	Methylation	GDSC	-0.1106904	0.00213004	-0.279993097	7.39*E* − 16
Methotrexate	mRNA	TUBA1C	Methylation	GDSC	-0.09871744	0.005699643	-0.271395469	4.70*E* − 15
OSI-930	mRNA	TUBA1C	Methylation	GDSC	-0.1095637	0.002316161	-0.271318342	6.18*E* − 15
Tivozanib	mRNA	TUBA1C	Methylation	GDSC	-0.1119652	0.001993638	-0.267389443	7.90*E* − 15

## Data Availability

The data used for this study were provided by public databases, which are included in this article.

## Supplementary Materials

Supplementary 1 Supplementary Figure 1: (a-h) the DSS curses of TUBA1C in BRCA, KIRC, LGG, LIHC, MESO, PAAD, SKCM, and UCEC. Which demonstrated that elevated expression of TUBA1C mRNA is correlated with unfavorable prognosis of patients with UCEC, SKCM, PAAD, MESO, LIHC, LGG, and KIRC. (i) The cox regression analysis for DSS and TUBA1C expression in 33 tumours based on TCGA database. DSS: disease specific survival.

Supplementary 2 Supplementary Figure 2: (a-c) the DFI curses of TUBA1C in LUAD, PAAD, and SARC. The high expression of TUBA1C mRNA is correlated to the unfavorable prognosis in PAAD, LUAD, and SARC. (d) The cox regression analysis for DFI and TUBA1C expression in 33 tumours based on TCGA database. DFI: disease free interval.

Supplementary 3 Supplementary Figure 3: (a-j) the PFI curses of TUBA1C in ACC, GBM, KICH, KIRC, LGG, LUAD, MESO, PAAD, READ, and SARC. The high expression of TUBA1C mRNA is correlated to the unfavorable prognosis in SARC, PAAD, MESO, LUAD, LGG, KIRC, KICH, GBM, and ACC, while in READ, the low expression of TUBA1C mRNA is associated with unfavorable prognosis. (k) The cox regression analysis for PFI and TUBA1C expression in 33 tumours based on TCGA database. PFI: progression free interval.

Supplementary 4 Supplementary Figure 4: (a-f) the OS curses of the TUBA1C CNV in BRCA, KICH, KIRC, KIRP, LUAD, and UCEC. (g-m) The PFS curses of the TUBA1C CNV in BRCA, KICH, KIRC, KIRP, LUAD, MESO, and UCS. The OS of TUBA1C wild type was remarkably improved as opposed to that of deletion/amplification type, and in BRCA, KICH, KIRC, KIRP, LUAD, MESO, and UCS, the PFS of TUBA1C wild type was also remarkably improved as opposed to that of deletion/amplification type.

Supplementary 5 Supplementary Figure 5: (a-d) the correlation between TUBA1C and the immune score in BRCA, OV, PCPG, and UCEC. (e-j) The correlation between TUBA1C and the stromal score in BRCA, COAD, ESCA, STAD, UCEC, and THCA. It can be seen that TUBA1C mRNA expression and the correlation score are negatively correlated in UCEC, BRCA, COAD, ESCA, STAD, and UCEC.

Supplementary 6 Supplementary Figure 6: the correlation between TUBA1C mRNA and CYT.

Supplementary 7 Supplementary Table 1: cancer abbreviations in the TCGA database.

Supplementary 8 Supplementary Table 2: we counted the information of tumour patients collected in the TCGA database into supplementary Table 2, including the sample size, age, gender, and stage of patients.

Supplementary 9 Supplementary Table 3: the detailed information on the CNV types in each cancer type. a_total: the total amplification ratio; d_total: the total deletion ratio; a_hete: the heterozygous amplification ratio; d_hete: the heterozygous deletion ratio; a_homo: the homozygous amplification ratio; d_homo: the homozygous deletion ratio; Entrez: the number of cases included.

Supplementary 10 Supplementary Table 4: the association between TUBA1C CNV and TUBA1C mRNA expression in different cancers. Spm: Spearman rank correlation coefficient; fdr: false discovery rate; Entrez: the number of cases included.

Supplementary 11 Supplementary Table 5: the correlation between methylation and mRNA expression in different cancers. Spm: Spearman rank correlation coefficient; fdr: false discovery rate; Entrez: the number of cases included.
