# Supplementary material for: Comprehensive Multiomic Analysis Identified TUBA1C as a Potential Prognostic Biological Marker of Immune-Related Therapy in Pan-Cancer
Source: Comput Math Methods Med. 2022 Oct 30;2022:9493115. doi: 10.1155/2022/9493115 (PMC9713470; doi:10.1155/2022/9493115)
Supplement: Supplementary 1 — Supplementary Figure 1: (a-h) the DSS curses of TUBA1C in BRCA, KIRC, LGG, LIHC, MESO, PAAD, SKCM, and UCEC. Which demonstrated that elevated expression of TUBA1C mRNA is correlated with unfavorable prognosis of patients with UCEC, SKCM, PAAD, MESO, LIHC, LGG, and KIRC. (i) The cox regression analysis for DSS and TUBA1C expression in 33 tumours based on TCGA database. DSS: disease specific survival. [file 9493115.f1.pdf]

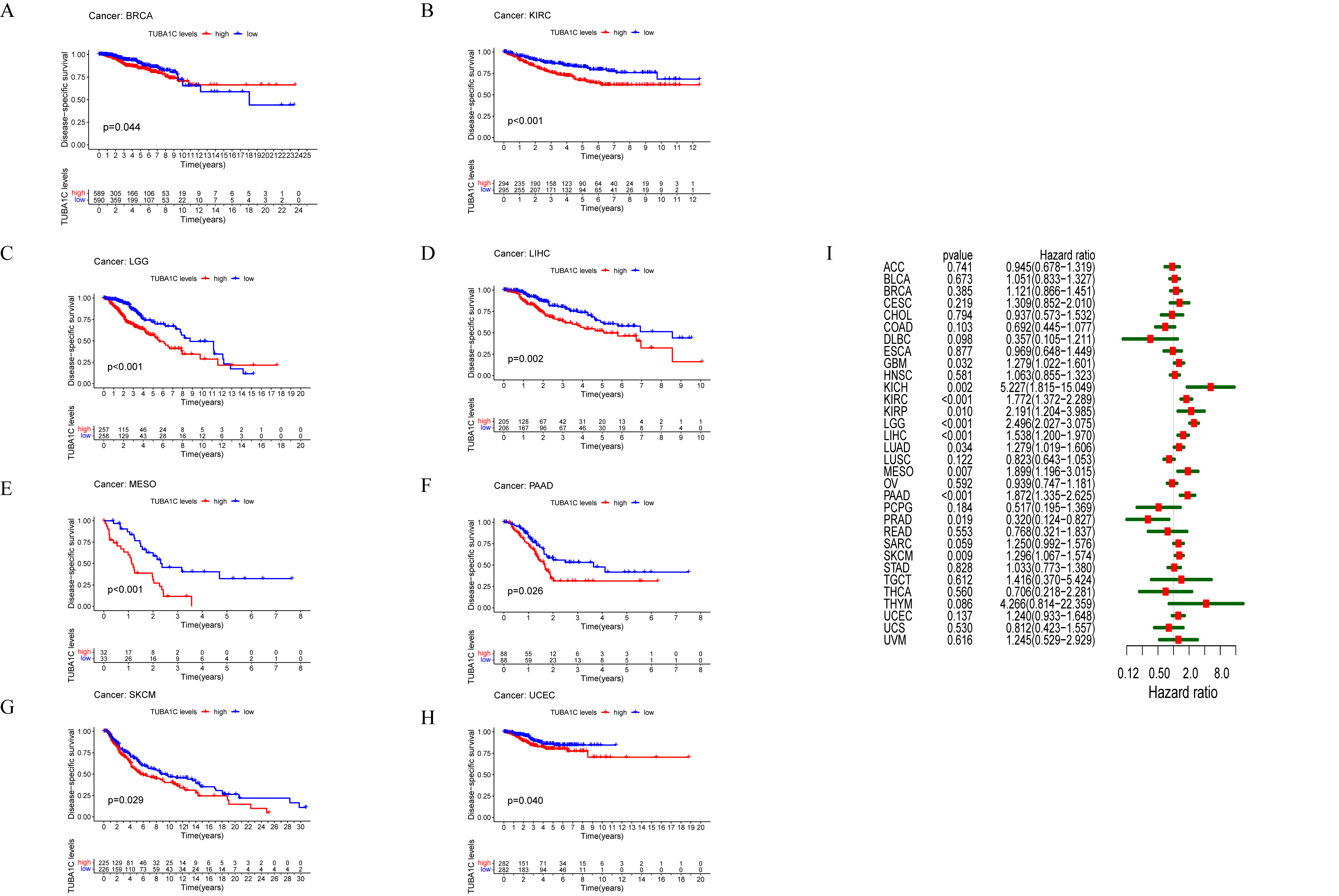
A
Cancer: BRCA
TUBA1C levels high low
Disease−specific survival
p=0.044
Time(years)
TUBA1C levels
high 589 305 166 106 53 19 9 7 6 5 3 1 0
low 590 359 199 107 53 22 10 7 5 4 3 2 0
B
Cancer: KIRC
TUBA1C levels high low
Disease−specific survival
p<0.001
Time(years)
TUBA1C levels
high 294 235 190 158 123 90 64 40 24 19 9 3 1
low 295 255 207 171 132 94 65 41 26 19 9 2 1
C
Cancer: LGG
TUBA1C levels high low
Disease−specific survival
p<0.001
Time(years)
TUBA1C levels
high 257 115 46 24 8 5 3 2 1 0 0
low 258 129 43 28 16 12 6 3 0 0 0
D
Cancer: LIHC
TUBA1C levels high low
Disease−specific survival
p=0.002
Time(years)
TUBA1C levels
high 205 128 67 42 31 20 13 4 2 1 1
low 206 167 96 67 46 30 19 8 7 4 0
E
Cancer: MESO
TUBA1C levels high low
Disease−specific survival
p<0.001
Time(years)
TUBA1C levels
high 32 17 8 2 0 0 0 0 0
low 33 26 16 9 6 4 2 1 0
F
Cancer: PAAD
TUBA1C levels high low
Disease−specific survival
p=0.026
Time(years)
TUBA1C levels
high 88 55 12 6 3 3 1 0 0
low 88 59 23 13 8 5 1 1 0
G
Cancer: SKCM
TUBA1C levels high low
Disease−specific survival
p=0.029
Time(years)
TUBA1C levels
high 225 129 81 46 32 25 14 9 6 5 3 3 2 0 0 0
low 226 159 110 73 59 43 34 24 16 14 7 4 4 4 4 2
H
Cancer: UCEC
TUBA1C levels high low
Disease−specific survival
p=0.040
Time(years)
TUBA1C levels
high 282 151 71 34 15 6 3 2 1 1 0
low 282 183 94 46 11 1 0 0 0 0 0
I
pvalue Hazard ratio
ACC 0.741 0.945(0.678−1.319)
BLCA 0.673 1.051(0.833−1.327)
BRCA 0.385 1.121(0.866−1.451)
CESC 0.219 1.309(0.852−2.010)
CHOL 0.794 0.937(0.573−1.532)
COAD 0.103 0.692(0.445−1.077)
DLBC 0.098 0.357(0.105−1.211)
ESCA 0.877 0.969(0.648−1.449)
GBM 0.032 1.279(1.022−1.601)
HNSC 0.581 1.063(0.855−1.323)
KICH 0.002 5.227(1.815−15.049)
KIRC <0.001 1.772(1.372−2.289)
KIRP 0.010 2.191(1.204−3.985)
LGG <0.001 2.496(2.027−3.075)
LIHC <0.001 1.538(1.200−1.970)
LUAD 0.034 1.279(1.019−1.606)
LUSC 0.122 0.823(0.643−1.053)
MESO 0.007 1.899(1.196−3.015)
OV 0.592 0.939(0.747−1.181)
PAAD <0.001 1.872(1.335−2.625)
PCPG 0.184 0.517(0.195−1.369)
PRAD 0.019 0.320(0.124−0.827)
READ 0.553 0.768(0.321−1.837)
SARC 0.059 1.250(0.992−1.576)
SKCM 0.009 1.296(1.067−1.574)
STAD 0.828 1.033(0.773−1.380)
TGCT 0.612 1.416(0.370−5.424)
THCA 0.560 0.706(0.218−2.281)
THYM 0.086 4.266(0.814−22.359)
UCEC 0.137 1.240(0.933−1.648)
UCS 0.530 0.812(0.423−1.557)
UVM 0.616 1.245(0.529−2.929)
0.12 0.50 2.0 8.0
Hazard ratio
