# Supplementary material for: Comprehensive Multiomic Analysis Identified TUBA1C as a Potential Prognostic Biological Marker of Immune-Related Therapy in Pan-Cancer
Source: Comput Math Methods Med. 2022 Oct 30;2022:9493115. doi: 10.1155/2022/9493115 (PMC9713470; doi:10.1155/2022/9493115)
Supplement: Supplementary 2 — Supplementary Figure 2: (a-c) the DFI curses of TUBA1C in LUAD, PAAD, and SARC. The high expression of TUBA1C mRNA is correlated to the unfavorable prognosis in PAAD, LUAD, and SARC. (d) The cox regression analysis for DFI and TUBA1C expression in 33 tumours based on TCGA database. DFI: disease free interval. [file 9493115.f2.pdf]

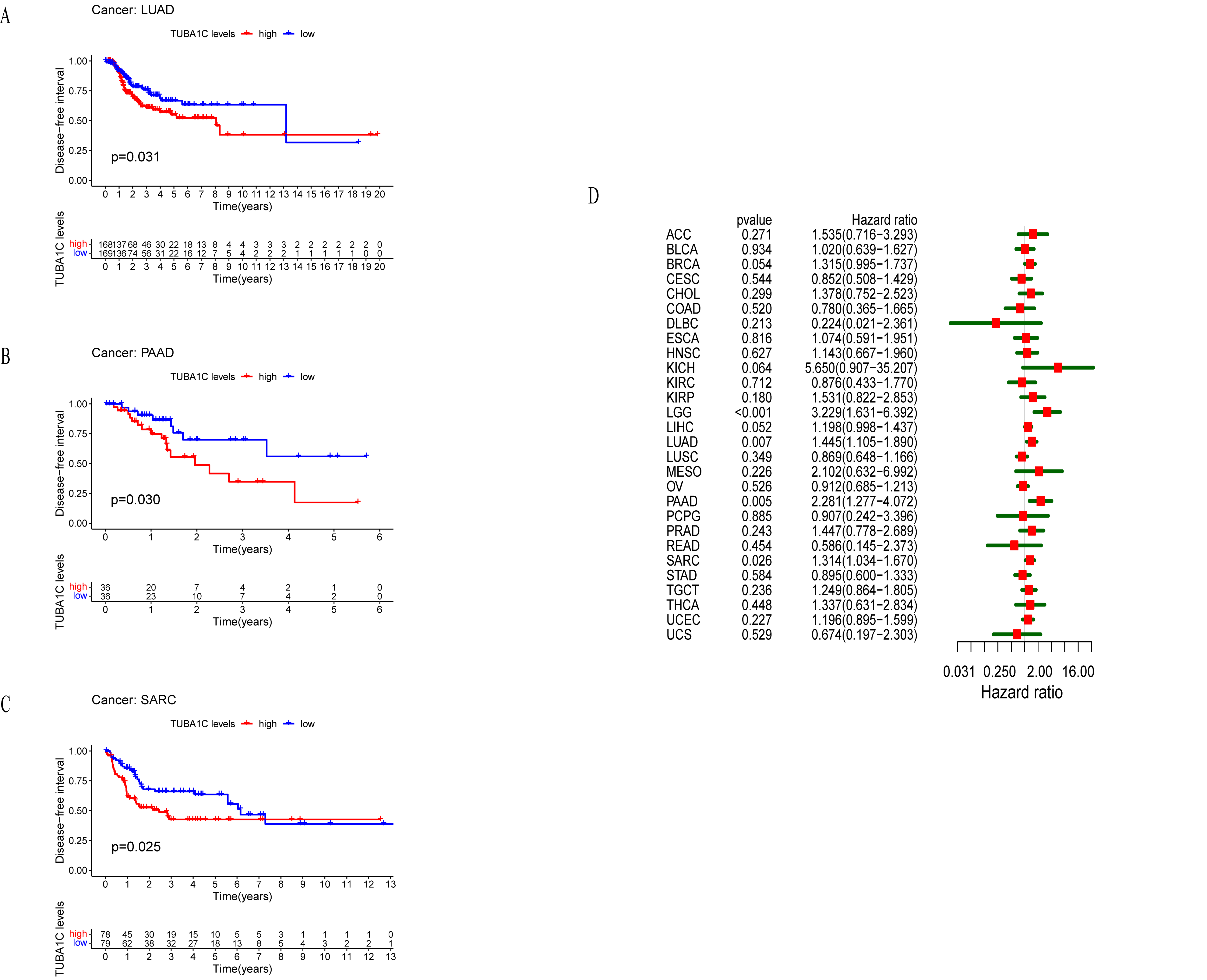
A
Cancer: LUAD
TUBA1C levels high low
Disease−free interval
1.00
0.75
0.50
0.25
0.00
p=0.031
0 1 2 3 4 5 6 7 8 9 10 11 12 13 14 15 16 17 18 19 20
Time(years)
TUBA1C levels
high 168137 68 46 30 22 18 13 8 4 4 3 3 2 2 2 2 2 2 2 0
low 169136 74 56 31 22 16 12 7 5 4 2 2 2 1 1 1 1 1 0 0
0 1 2 3 4 5 6 7 8 9 10 11 12 13 14 15 16 17 18 19 20
Time(years)
B
Cancer: PAAD
TUBA1C levels high low
Disease−free interval
1.00
0.75
0.50
0.25
0.00
p=0.030
0 1 2 3 4 5 6
Time(years)
TUBA1C levels
high 36 20 7 4 2 1 0
low 36 23 10 7 4 2 0
0 1 2 3 4 5 6
Time(years)
C
Cancer: SARC
TUBA1C levels high low
Disease−free interval
1.00
0.75
0.50
0.25
0.00
p=0.025
0 1 2 3 4 5 6 7 8 9 10 11 12 13
Time(years)
TUBA1C levels
high 78 45 30 19 15 10 5 5 3 1 1 1 1 0
low 79 62 38 32 27 18 13 8 5 4 3 2 2 1
0 1 2 3 4 5 6 7 8 9 10 11 12 13
Time(years)
D
pvalue Hazard ratio
ACC 0.271 1.535(0.716−3.293)
BLCA 0.934 1.020(0.639−1.627)
BRCA 0.054 1.315(0.995−1.737)
CESC 0.544 0.852(0.508−1.429)
CHOL 0.299 1.378(0.752−2.523)
COAD 0.520 0.780(0.365−1.665)
DLBC 0.213 0.224(0.021−2.361)
ESCA 0.816 1.074(0.591−1.951)
HNSC 0.627 1.143(0.667−1.960)
KICH 0.064 5.650(0.907−35.207)
KIRC 0.712 0.876(0.433−1.770)
KIRP 0.180 1.531(0.822−2.853)
LGG <0.001 3.229(1.631−6.392)
LIHC 0.052 1.198(0.998−1.437)
LUAD 0.007 1.445(1.105−1.890)
LUSC 0.349 0.869(0.648−1.166)
MESO 0.226 2.102(0.632−6.992)
OV 0.526 0.912(0.685−1.213)
PAAD 0.005 2.281(1.277−4.072)
PCPG 0.885 0.907(0.242−3.396)
PRAD 0.243 1.447(0.778−2.689)
READ 0.454 0.586(0.145−2.373)
SARC 0.026 1.314(1.034−1.670)
STAD 0.584 0.895(0.600−1.333)
TGCT 0.236 1.249(0.864−1.805)
THCA 0.448 1.337(0.631−2.834)
UCEC 0.227 1.196(0.895−1.599)
UCS 0.529 0.674(0.197−2.303)
0.031 0.250 2.00 16.00
Hazard ratio
