# Supplementary material for: Comprehensive Multiomic Analysis Identified TUBA1C as a Potential Prognostic Biological Marker of Immune-Related Therapy in Pan-Cancer
Source: Comput Math Methods Med. 2022 Oct 30;2022:9493115. doi: 10.1155/2022/9493115 (PMC9713470; doi:10.1155/2022/9493115)
Supplement: Supplementary 3 — Supplementary Figure 3: (a-j) the PFI curses of TUBA1C in ACC, GBM, KICH, KIRC, LGG, LUAD, MESO, PAAD, READ, and SARC. The high expression of TUBA1C mRNA is correlated to the unfavorable prognosis in SARC, PAAD, MESO, LUAD, LGG, KIRC, KICH, GBM, and ACC, while in READ, the low expression of TUBA1C mRNA is associated with unfavorable prognosis. (k) The cox regression analysis for PFI and TUBA1C expression in 33 tumours based on TCGA database. PFI: progression free interval. [file 9493115.f3.pdf]

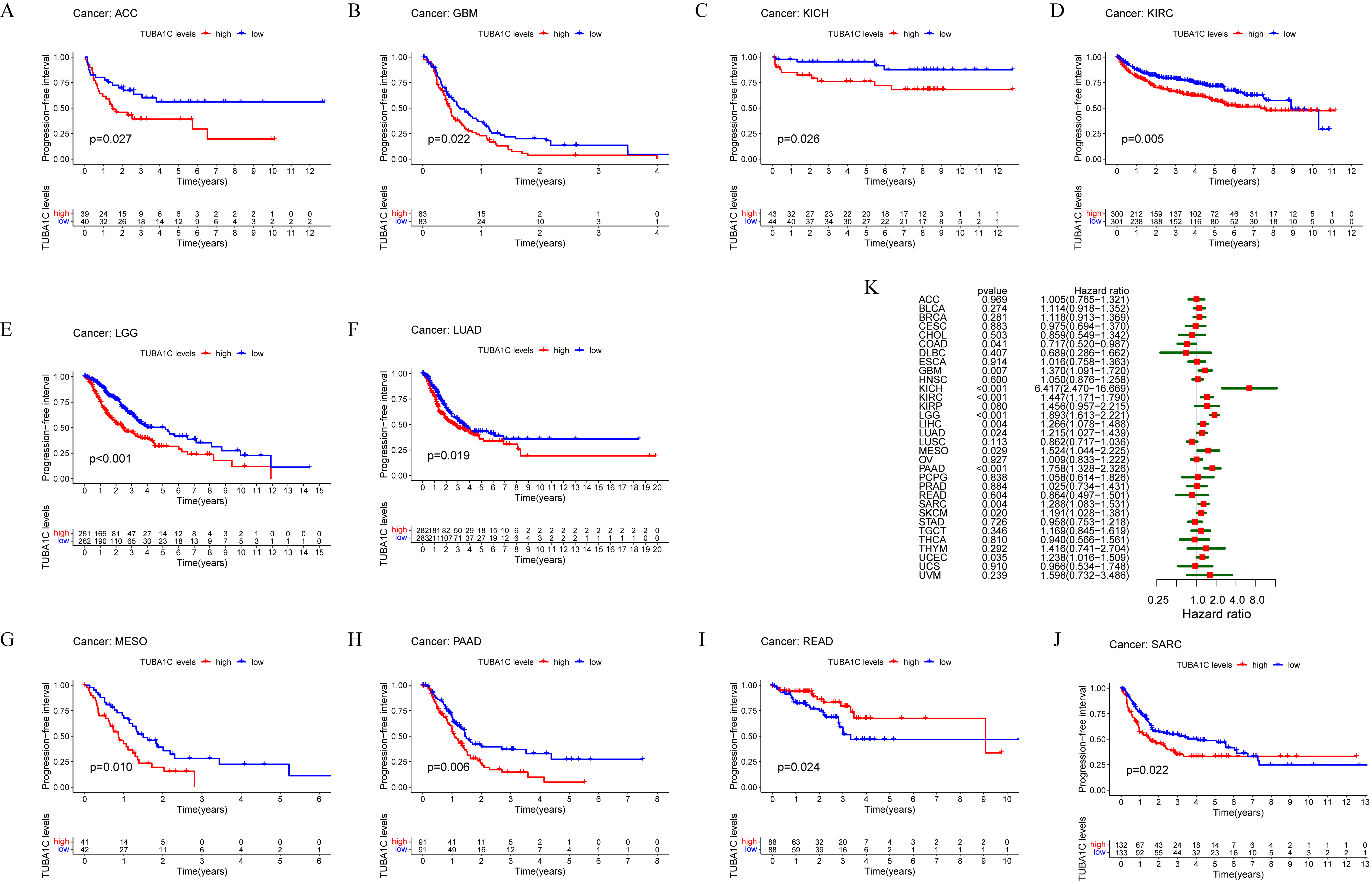

A
Cancer: ACC
p=0.027
B
Cancer: GBM
p=0.022
C
Cancer: KICH
p=0.026
D
Cancer: KIRC
p=0.005
E
Cancer: LGG
p<0.001
F
Cancer: LUAD
p=0.019
G
Cancer: MESO
p=0.010
H
Cancer: PAAD
p=0.006
I
Cancer: READ
p=0.024
J
Cancer: SARC
p=0.022
TUBA1C levels high low
Progression−free interval
Time(years)
K
pvalue
Hazard ratio
ACC 0.969 1.005(0.765−1.321)
BLCA 0.274 1.114(0.918−1.352)
BRCA 0.281 1.118(0.913−1.369)
CESC 0.883 0.975(0.694−1.370)
CHOL 0.503 0.859(0.549−1.342)
COAD 0.041 0.717(0.520−0.987)
DLBC 0.407 0.689(0.286−1.662)
ESCA 0.914 1.016(0.758−1.363)
GBM 0.007 1.370(1.091−1.720)
HNSC 0.600 1.050(0.876−1.258)
KICH <0.001 6.417(2.470−16.669)
KIRC <0.001 1.447(1.171−1.790)
KIRP 0.080 1.456(0.957−2.215)
LGG <0.001 1.893(1.613−2.221)
LIHC 0.004 1.266(1.078−1.488)
LUAD 0.024 1.215(1.027−1.439)
LUSC 0.113 0.862(0.717−1.036)
MESO 0.029 1.524(1.044−2.225)
OV 0.927 1.009(0.833−1.222)
PAAD <0.001 1.758(1.328−2.326)
PCPG 0.838 1.058(0.614−1.826)
PRAD 0.884 1.025(0.734−1.431)
READ 0.604 0.864(0.497−1.501)
SARC 0.004 1.288(1.083−1.531)
SKCM 0.020 1.191(1.028−1.381)
STAD 0.726 0.958(0.753−1.218)
TGCT 0.346 1.169(0.845−1.619)
THCA 0.810 0.940(0.566−1.561)
THYM 0.292 1.416(0.741−2.704)
UCEC 0.035 1.238(1.016−1.509)
UCS 0.910 0.966(0.534−1.748)
UVM 0.239 1.598(0.732−3.486)
0.25 1.0 2.0 4.0 8.0
Hazard ratio
