# Supplementary figures and images for: Comprehensive Multiomic Analysis Identified TUBA1C as a Potential Prognostic Biological Marker of Immune-Related Therapy in Pan-Cancer
Source: Comput Math Methods Med. 2022 Oct 30;2022:9493115. doi: 10.1155/2022/9493115 (PMC9713470; doi:10.1155/2022/9493115)

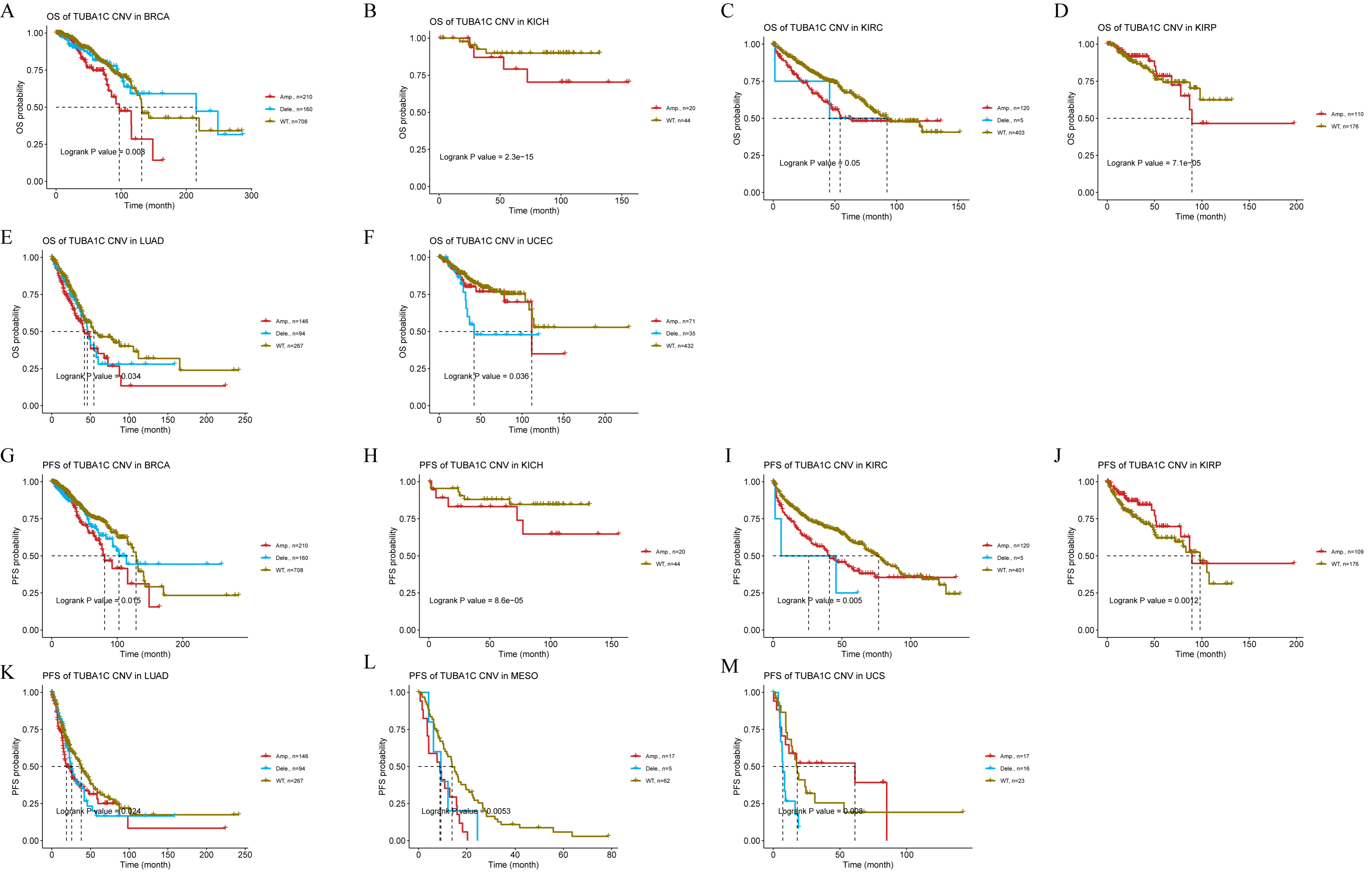

Supplement: Supplementary 4 — Supplementary Figure 4: (a-f) the OS curses of the TUBA1C CNV in BRCA, KICH, KIRC, KIRP, LUAD, and UCEC. (g-m) The PFS curses of the TUBA1C CNV in BRCA, KICH, KIRC, KIRP, LUAD, MESO, and UCS. The OS of TUBA1C wild type was remarkably improved as opposed to that of deletion/amplification type, and in BRCA, KICH, KIRC, KIRP, LUAD, MESO, and UCS, the PFS of TUBA1C wild type was also remarkably improved as opposed to that of deletion/amplification type. [file 9493115.f4.pdf]

A

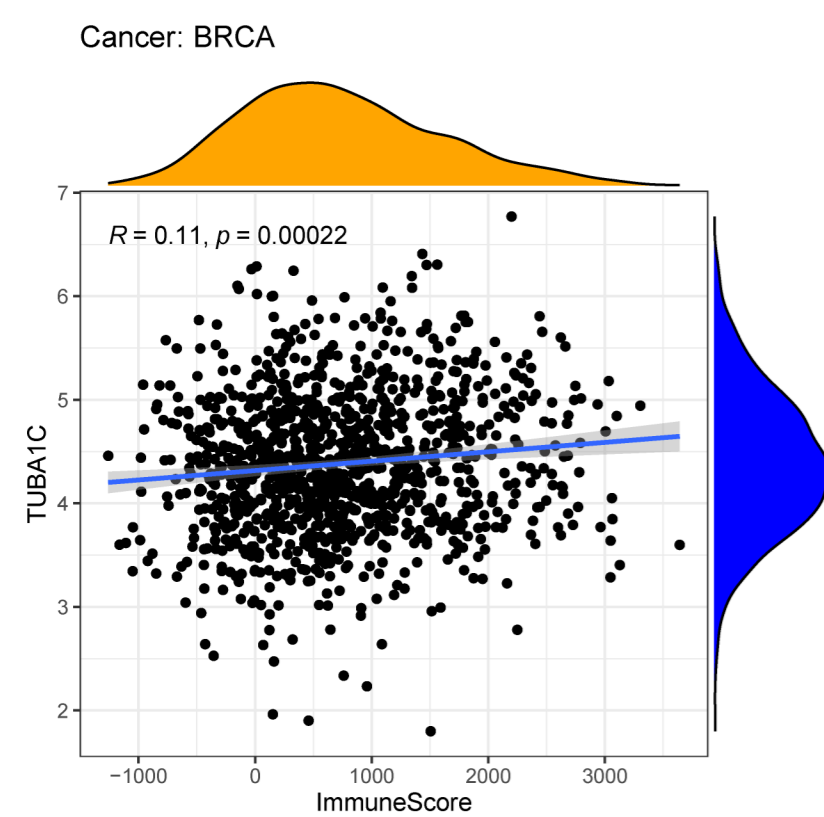

B

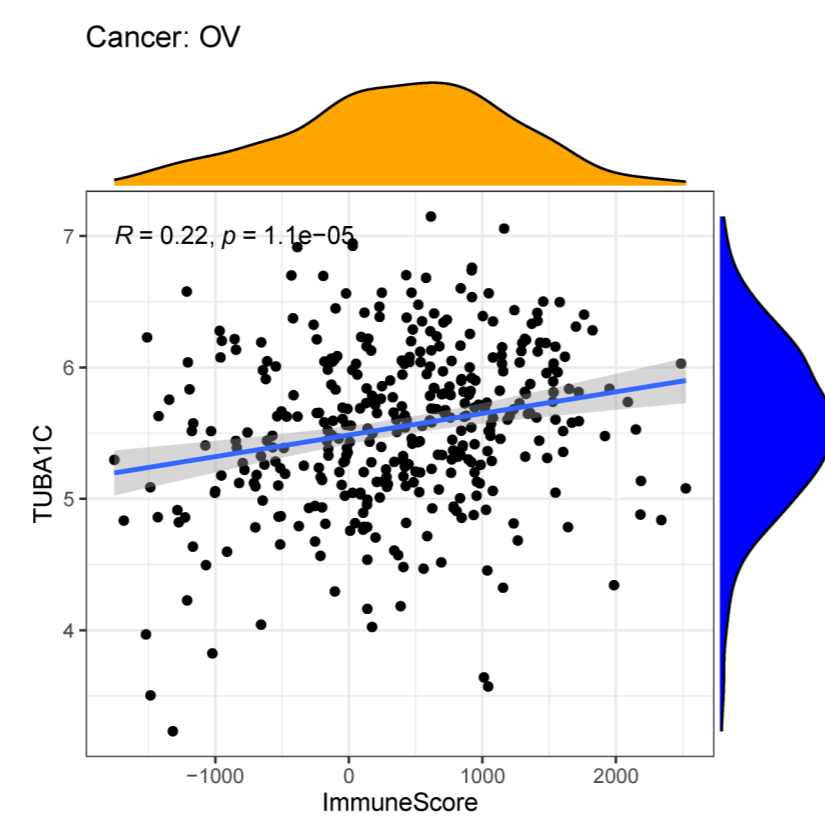

C

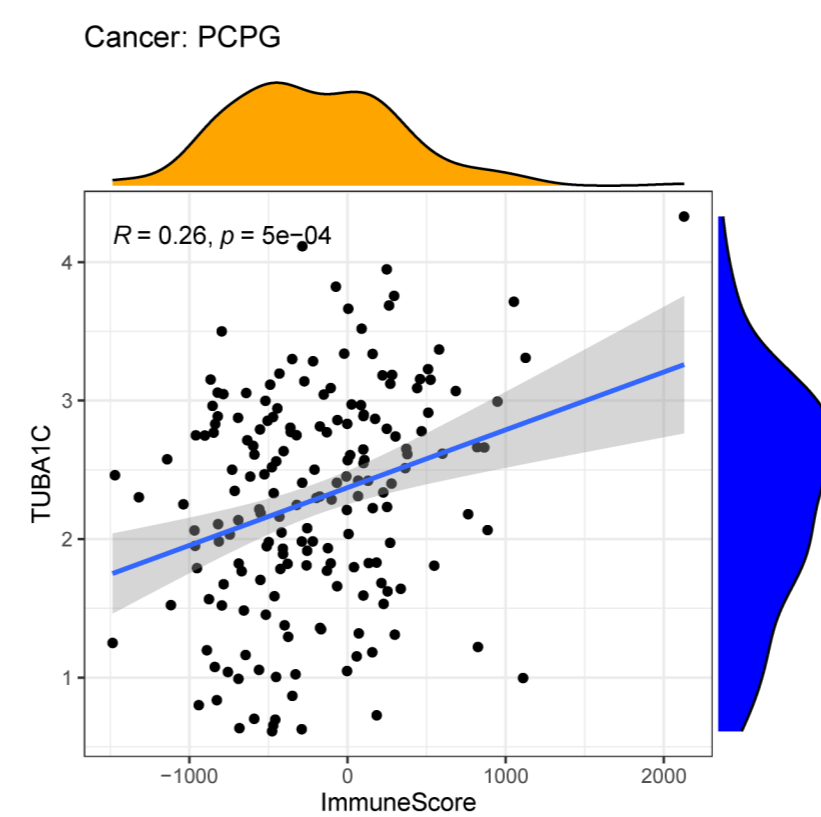

D

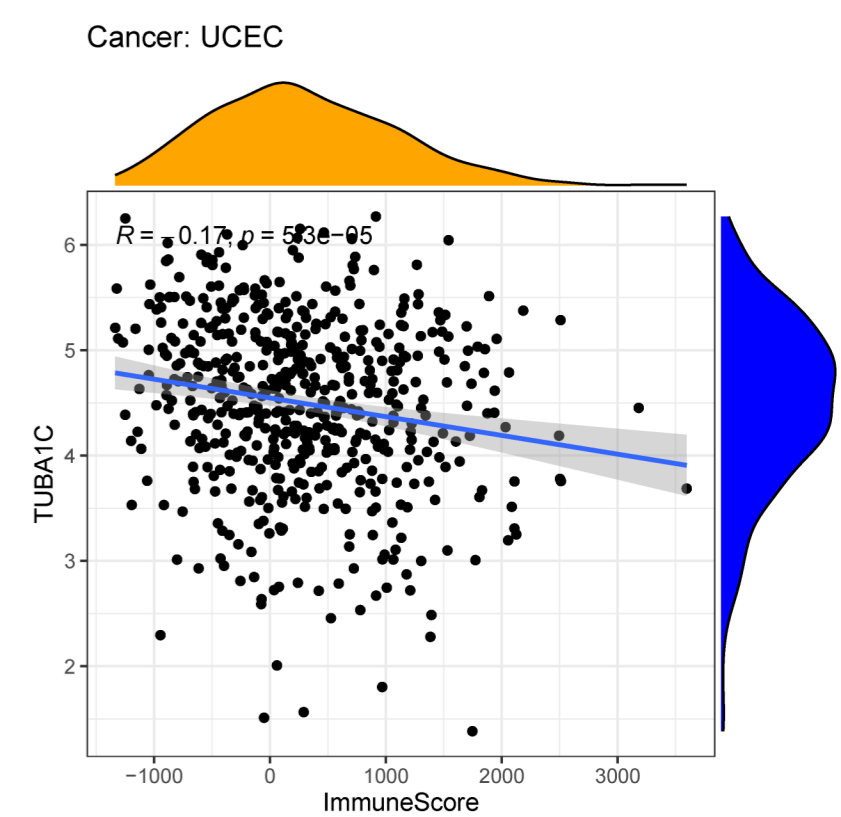

E

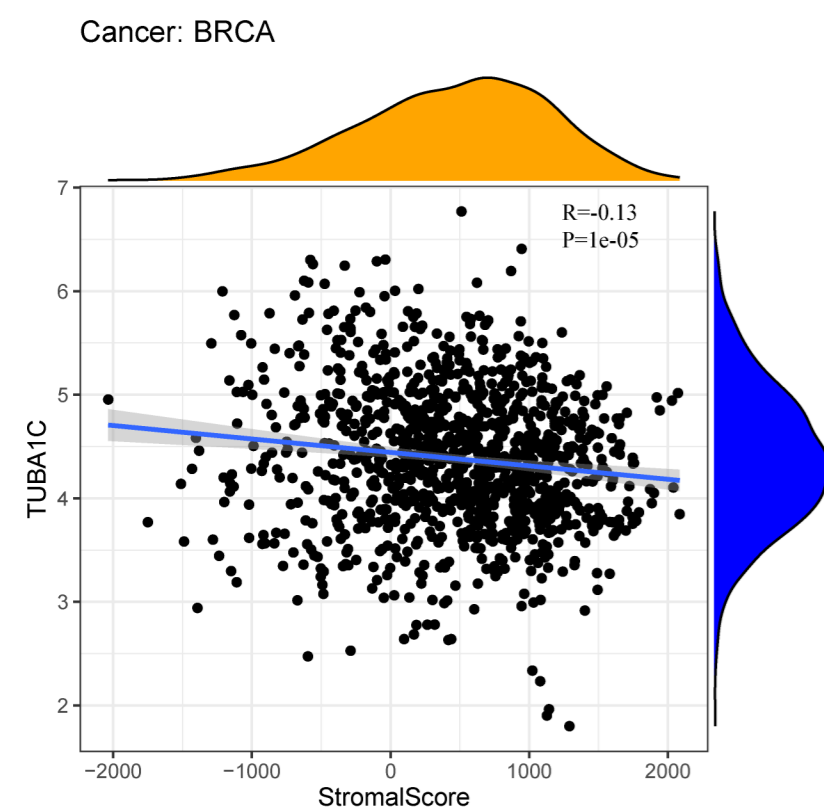

F

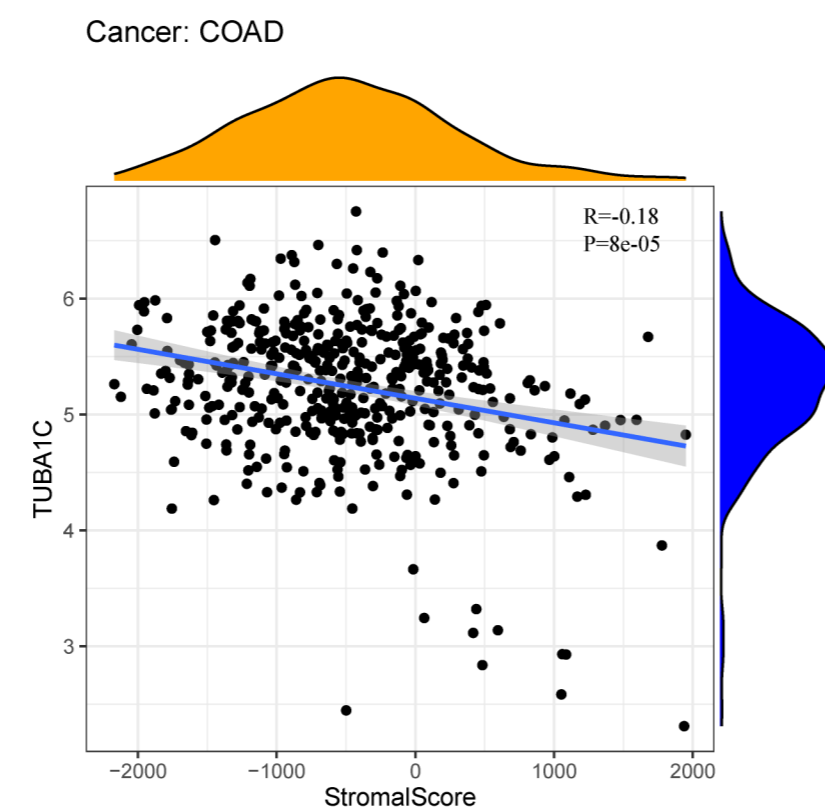

G

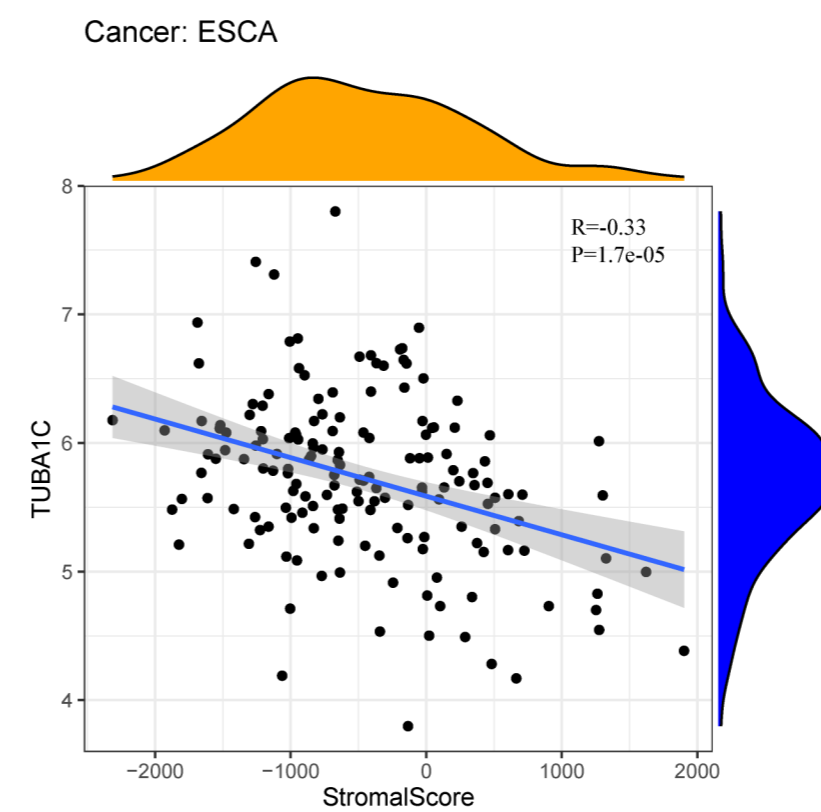

H

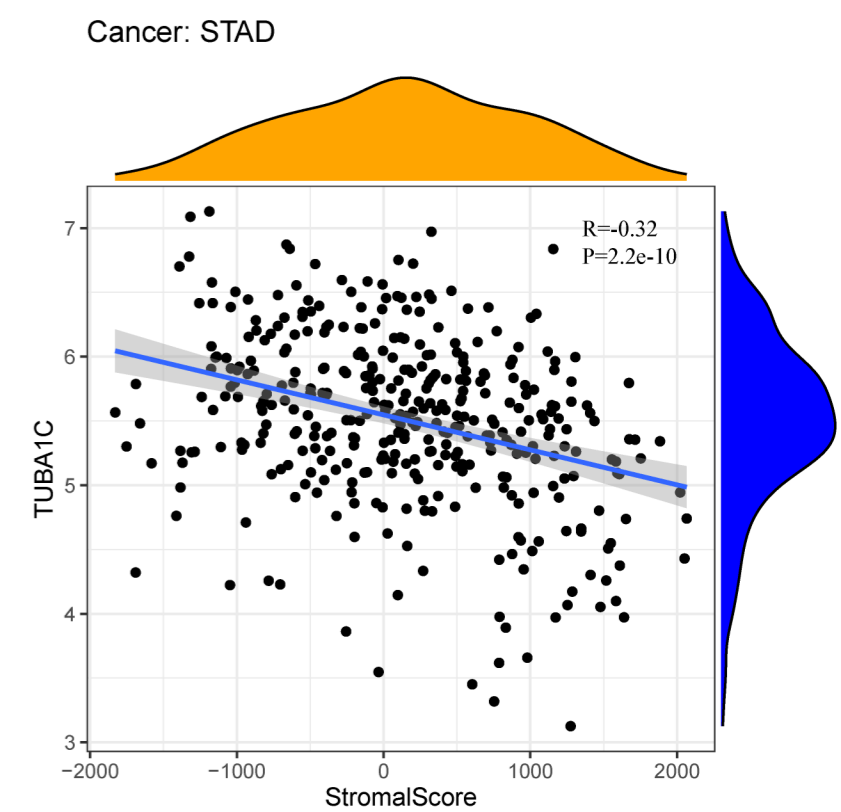

I

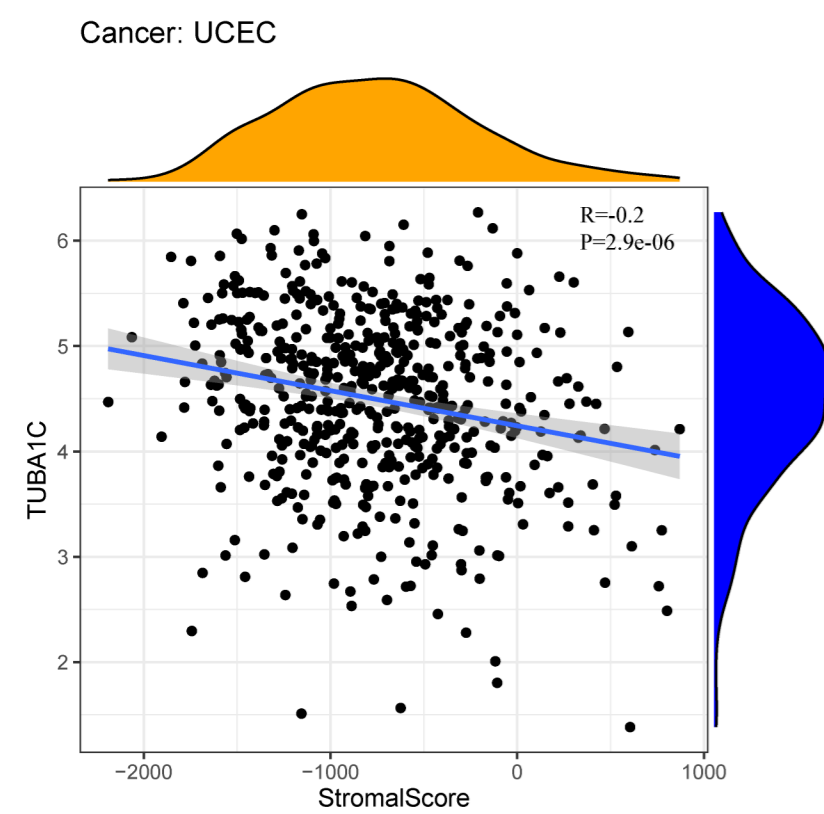

J

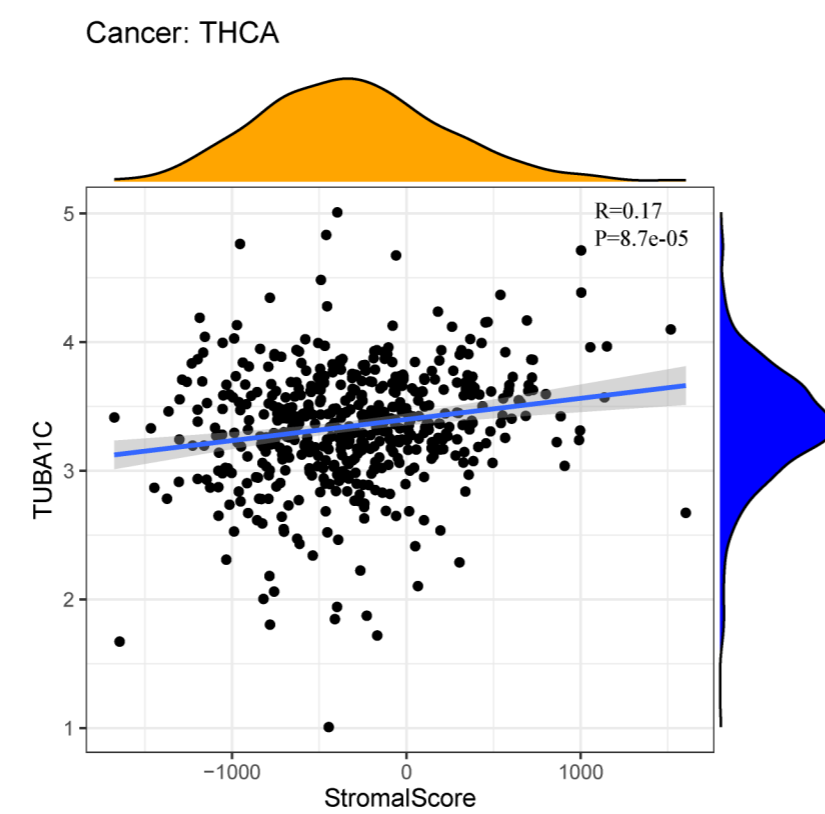

Supplement: Supplementary 5 — Supplementary Figure 5: (a-d) the correlation between TUBA1C and the immune score in BRCA, OV, PCPG, and UCEC. (e-j) The correlation between TUBA1C and the stromal score in BRCA, COAD, ESCA, STAD, UCEC, and THCA. It can be seen that TUBA1C mRNA expression and the correlation score are negatively correlated in UCEC, BRCA, COAD, ESCA, STAD, and UCEC. [file 9493115.f5.pdf]

# CYT

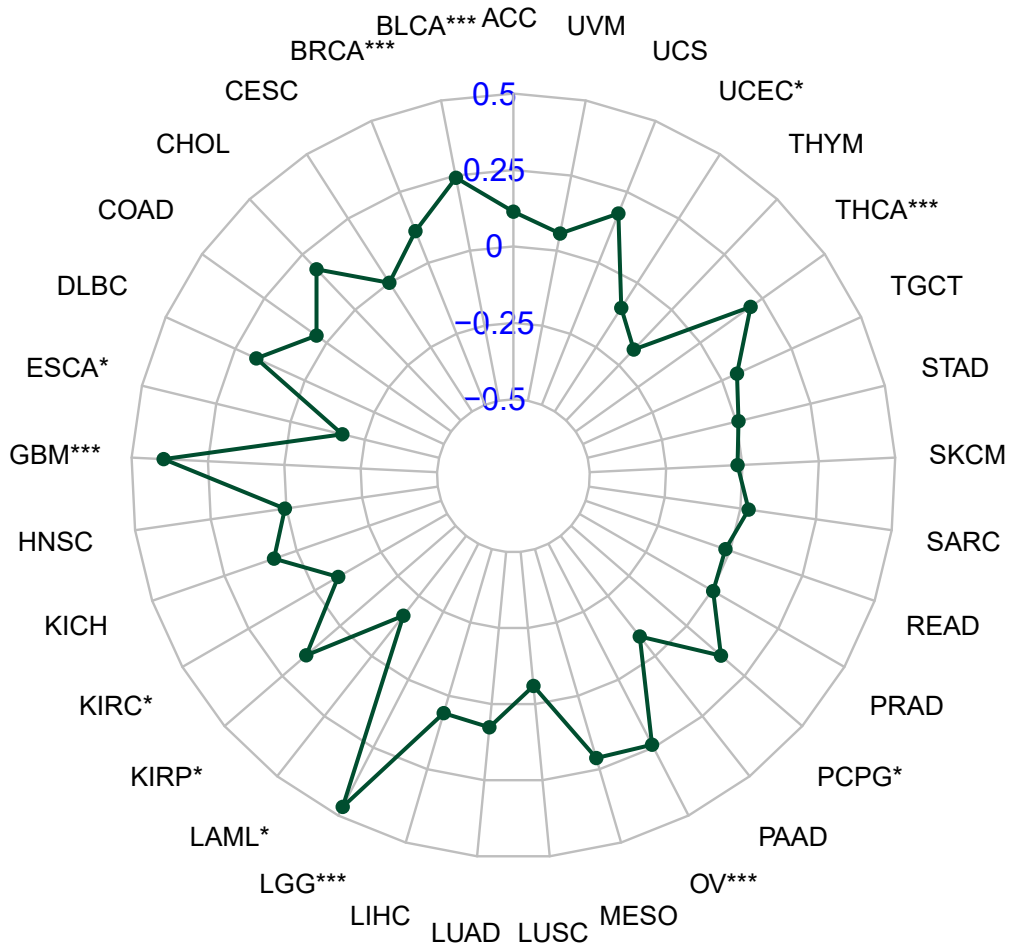

Supplement: Supplementary 6 — Supplementary Figure 6: the correlation between TUBA1C mRNA and CYT. [file 9493115.f6.pdf]
