# Supplementary material for: Comprehensive Multiomic Analysis Identified TUBA1C as a Potential Prognostic Biological Marker of Immune-Related Therapy in Pan-Cancer
Source: Comput Math Methods Med. 2022 Oct 30;2022:9493115. doi: 10.1155/2022/9493115 (PMC9713470; doi:10.1155/2022/9493115)
Supplement: Supplementary 8 — Supplementary Table 2: we counted the information of tumour patients collected in the TCGA database into supplementary Table 2, including the sample size, age, gender, and stage of patients. [file 9493115.f8.pdf]

Cancers	Tumor Samples	Age		Sex		Stage				genes
		<=65	>65	male	female	I	II	III	IV	
ACC	93	82	11	32	61	9	44	19	19	56537
BLCA	415	164	251	305	110	2	131	142	237	56537
BRCA	1103	782	320	12	1091	183	625	251	20	56537
CESC	309	274	35	0	309	-	-	-	-	56537
CHOL	36	17	19	16	20	16	8	1	7	56537
COAD	460	192	268	242	218	90	165	130	64	56537
DLBC	48	33	15	22	26	-	-	-	-	56537
ESCA	186	114	72	159	27	18	79	56	9	56537
GBM	601	417	184	367	50	-	-	-	-	56537
HNSC	530	346	183	387	143	27	74	82	272	56537
KICH	66	49	17	38	28	20	24	13	9	56537
KIRC	537	352	185	347	190	269	57	124	87	56537
KIRP	292	180	110	214	78	173	22	52	15	56537
LAML	200	144	56	109	91	-	-	-	-	56537
LGG	529	496	33	291	238	-	-	-	-	56537
LIHC	379	237	142	257	122	176	88	85	5	56537
LUAD	521	241	261	243	278	280	123	84	26	56537
LUSC	504	190	305	373	131	245	163	85	7	56537
MESO	87	47	40	71	16	10	16	45	16	56537
OV	600	418	182	-	600	-	-	-	-	56537
PAAD	186	96	90	103	83	21	152	4	6	56537
PCPG	184	162	22	82	102	-	-	-	-	56537
PRAD	499	356	143	499	-	-	-	-	-	56537
READ	167	83	84	91	76	30	51	51	25	56537
SARC	265	163	102	120	145	-	-	-	-	56537
SKCM	477	305	164	294	183	78	141	137	24	56537
STAD	443	197	241	285	158	59	130	183	44	56537
TGCT	139	137	2	139	-	56	12	14	-	56537
THCA	515	443	72	140	375	290	52	114	57	56537
THYM	124	81	42	60	64	-	-	-	-	56537
UCEC	548	308	237	-	548	-	-	-	-	56537
UCS	57	22	35	-	57	-	-	-	-	56537
UVM	80	46	34	35	45	0	39	36	4	56537
