# Supplementary material for: Comprehensive Multiomic Analysis Identified TUBA1C as a Potential Prognostic Biological Marker of Immune-Related Therapy in Pan-Cancer
Source: Comput Math Methods Med. 2022 Oct 30;2022:9493115. doi: 10.1155/2022/9493115 (PMC9713470; doi:10.1155/2022/9493115)
Supplement: Supplementary 9 — Supplementary Table 3: the detailed information on the CNV types in each cancer type. a_total: the total amplification ratio; d_total: the total deletion ratio; a_hete: the heterozygous amplification ratio; d_hete: the heterozygous deletion ratio; a_homo: the homozygous amplification ratio; d_homo: the homozygous deletion ratio; Entrez: the number of cases included. [file 9493115.f9.doc]

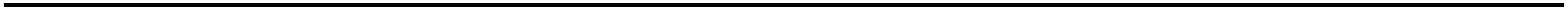

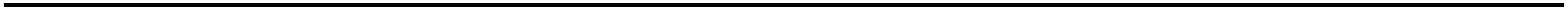

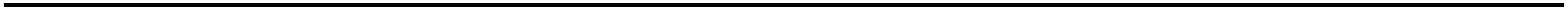
cancertype symbol a_total d_total a_hete d_hete a_homo d_homo entrez

ACC TUBA1C 74.44 1.11 72.22 1.11 2.22 0.00 84790.00

BLCA TUBA1C 21.32 16.42 20.83 16.18 0.49 0.25 84790.00

BRCA TUBA1C 19.44 14.91 18.98 14.91 0.46 0.00 84790.00

CESC TUBA1C 17.63 5.42 17.63 5.42 0.00 0.00 84790.00

CHOL TUBA1C 25.00 8.33 22.22 8.33 2.78 0.00 84790.00

COAD TUBA1C 21.29 10.20 21.29 10.20 0.00 0.00 84790.00

DLBC TUBA1C 20.83 6.25 16.67 4.17 4.17 2.08 84790.00

ESCA TUBA1C 19.57 21.74 19.02 21.20 0.54 0.54 84790.00

GBM TUBA1C 9.01 12.48 8.84 12.31 0.17 0.17 84790.00

HNSC TUBA1C 16.28 10.54 16.28 10.54 0.00 0.00 84790.00

KICH TUBA1C 31.82 1.52 31.82 1.52 0.00 0.00 84790.00

KIRC TUBA1C 22.73 0.95 22.73 0.95 0.00 0.00 84790.00

KIRP TUBA1C 38.54 0.35 38.54 0.35 0.00 0.00 84790.00

LAML TUBA1C 0.00 1.05 0.00 0.52 0.00 0.52 84790.00

LGG TUBA1C 2.34 13.26 2.34 11.89 0.00 1.36 84790.00

LIHC TUBA1C 12.97 10.54 12.70 10.54 0.27 0.00 84790.00

LUAD TUBA1C 28.68 18.22 27.71 18.22 0.97 0.00 84790.00

LUSC TUBA1C 30.54 12.18 29.94 12.18 0.60 0.00 84790.00

MESO TUBA1C 19.54 5.75 19.54 5.75 0.00 0.00 84790.00

OV TUBA1C 34.02 17.79 32.99 17.62 1.04 0.17 84790.00

PAAD TUBA1C 12.50 19.02 12.50 18.48 0.00 0.54 84790.00

PCPG TUBA1C 7.41 1.85 6.79 1.85 0.62 0.00 84790.00

PRAD TUBA1C 3.66 4.67 3.05 4.67 0.61 0.00 84790.00

READ TUBA1C 15.15 10.30 15.15 10.30 0.00 0.00 84790.00

SARC TUBA1C 15.56 26.46 14.40 26.07 1.17 0.39 84790.00

SKCM TUBA1C 11.72 20.71 10.08 20.71 1.63 0.00 84790.00

STAD TUBA1C 19.50 11.34 19.27 11.11 0.23 0.23 84790.00

TGCT TUBA1C 63.33 0.00 62.67 0.00 0.67 0.00 84790.00

THCA TUBA1C 3.01 0.00 3.01 0.00 0.00 0.00 84790.00

THYM TUBA1C 3.25 5.69 3.25 5.69 0.00 0.00 84790.00

UCEC TUBA1C 13.17 6.49 12.06 6.49 1.11 0.00 84790.00

UCS TUBA1C 30.36 28.57 30.36 28.57 0.00 0.00 84790.00

UVM TUBA1C 3.75 2.50 3.75 2.50 0.00 0.00 84790.00
