# Supplementary material for: Comprehensive Multiomic Analysis Identified TUBA1C as a Potential Prognostic Biological Marker of Immune-Related Therapy in Pan-Cancer
Source: Comput Math Methods Med. 2022 Oct 30;2022:9493115. doi: 10.1155/2022/9493115 (PMC9713470; doi:10.1155/2022/9493115)
Supplement: Supplementary 10 — Supplementary Table 4: the association between TUBA1C CNV and TUBA1C mRNA expression in different cancers. Spm: Spearman rank correlation coefficient; fdr: false discovery rate; Entrez: the number of cases included. [file 9493115.f10.doc]

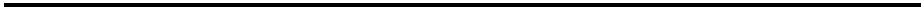

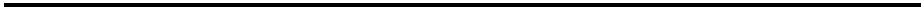

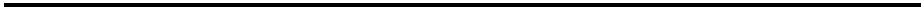
cancertype symbol spm fdr entrez

ACC TUBA1C 0.19 0.16 84790.00

BLCA TUBA1C 0.21 0.00 84790.00

BRCA TUBA1C 0.01 0.78 84790.00

CESC TUBA1C 0.17 0.01 84790.00

CHOL TUBA1C 0.42 0.04 84790.00

COAD TUBA1C 0.27 0.00 84790.00

DLBC TUBA1C 0.41 0.06 84790.00

ESCA TUBA1C 0.24 0.00 84790.00

GBM TUBA1C 0.12 0.24 84790.00

HNSC TUBA1C 0.11 0.02 84790.00

KICH TUBA1C 0.24 0.12 84790.00

KIRC TUBA1C 0.45 0.00 84790.00

KIRP TUBA1C 0.33 0.00 84790.00

LAML TUBA1C 0.15 0.32 84790.00

LGG TUBA1C 0.09 0.06 84790.00

LIHC TUBA1C 0.18 0.00 84790.00

LUAD TUBA1C 0.34 0.00 84790.00

LUSC TUBA1C 0.30 0.00 84790.00

MESO TUBA1C 0.32 0.01 84790.00

OV TUBA1C 0.25 0.00 84790.00

PAAD TUBA1C 0.13 0.13 84790.00

PCPG TUBA1C -0.01 0.95 84790.00

PRAD TUBA1C -0.03 0.66 84790.00

READ TUBA1C 0.31 0.01 84790.00

SARC TUBA1C 0.07 0.33 84790.00

SKCM TUBA1C 0.21 0.00 84790.00

STAD TUBA1C 0.28 0.00 84790.00

TGCT TUBA1C 0.19 0.04 84790.00

THCA TUBA1C 0.06 0.37 84790.00

THYM TUBA1C 0.36 0.00 84790.00

UCEC TUBA1C 0.06 0.54 84790.00

UCS TUBA1C 0.09 0.61 84790.00

UVM TUBA1C -0.11 0.52 84790.00
