# Supplementary material for: Comprehensive Multiomic Analysis Identified TUBA1C as a Potential Prognostic Biological Marker of Immune-Related Therapy in Pan-Cancer
Source: Comput Math Methods Med. 2022 Oct 30;2022:9493115. doi: 10.1155/2022/9493115 (PMC9713470; doi:10.1155/2022/9493115)
Supplement: Supplementary 11 — Supplementary Table 5: the correlation between methylation and mRNA expression in different cancers. Spm: Spearman rank correlation coefficient; fdr: false discovery rate; Entrez: the number of cases included. [file 9493115.f11.doc]

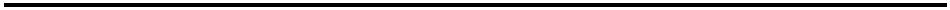

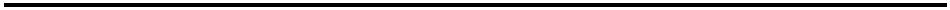

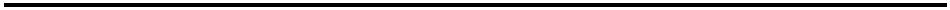
cancertype symbol spm fdr entrez

ACC TUBA1C -0.69 0.00 84790.00

BLCA TUBA1C -0.10 0.04 84790.00

BRCA TUBA1C -0.23 0.00 84790.00

CESC TUBA1C -0.14 0.01 84790.00

CHOL TUBA1C -0.38 0.02 84790.00

COAD TUBA1C -0.23 0.00 84790.00

DLBC TUBA1C -0.19 0.19 84790.00

ESCA TUBA1C -0.33 0.00 84790.00

GBM TUBA1C -0.70 0.00 84790.00

HNSC TUBA1C -0.18 0.00 84790.00

KICH TUBA1C -0.31 0.01 84790.00

KIRC TUBA1C -0.22 0.00 84790.00

KIRP TUBA1C -0.10 0.10 84790.00

LAML TUBA1C -0.21 0.01 84790.00

LGG TUBA1C -0.44 0.00 84790.00

LIHC TUBA1C -0.45 0.00 84790.00

LUAD TUBA1C -0.35 0.00 84790.00

LUSC TUBA1C -0.30 0.00 84790.00

MESO TUBA1C -0.31 0.00 84790.00

OV TUBA1C -0.82 0.01 84790.00

PAAD TUBA1C -0.54 0.00 84790.00

PCPG TUBA1C -0.48 0.00 84790.00

PRAD TUBA1C -0.62 0.00 84790.00

READ TUBA1C -0.40 0.00 84790.00

SARC TUBA1C -0.54 0.00 84790.00

SKCM TUBA1C -0.23 0.00 84790.00

STAD TUBA1C -0.32 0.00 84790.00

TGCT TUBA1C -0.49 0.00 84790.00

THCA TUBA1C -0.22 0.00 84790.00

THYM TUBA1C -0.15 0.09 84790.00

UCEC TUBA1C -0.22 0.00 84790.00

UCS TUBA1C -0.47 0.00 84790.00

UVM TUBA1C -0.61 0.00 84790.00
